# Differential Effect of Newly Isolated Phages Belonging to PB1-Like, phiKZ-Like and LUZ24-Like Viruses against Multi-Drug Resistant *Pseudomonas aeruginosa* under Varying Growth Conditions

**DOI:** 10.3390/v9110315

**Published:** 2017-10-27

**Authors:** Simone Latz, Alex Krüttgen, Helga Häfner, Eva Miriam Buhl, Klaus Ritter, Hans-Peter Horz

**Affiliations:** 1 Institute of Medical Microbiology, RWTH Aachen University Hospital, 52074 Aachen, Germany; slatz@ukaachen.de (S.L.); kruttgen@yahoo.com (A.K.); Ritter.SK@gmx.de (K.R.); 2 Department of Infection Control and Infectious Diseases, RWTH Aachen University Hospital, 52074 Aachen, Germany; hhaefner@ukaachen.de; 3 Electron Microscopy Facility, RWTH Aachen University Hospital, 52074 Aachen, Germany; ebuhl@ukaachen.de

**Keywords:** phage therapy, *Pseudomonas aeruginosa*, multi-drug resistance, PB1-like viruses, phiKZ-like viruses, LUZ24-like viruses

## Abstract

In this study, we characterize three phages (SL1 SL2, and SL4), isolated from hospital sewage with lytic activity against clinical isolates of multi-drug resistant *Pseudomonas aeruginosa* (MDR-PA). The host spectrum ranged from 41% to 54%, with all three phages together covering 79% of all tested clinical isolates. Genome analysis revealed that SL1 (65,849 bp, 91 open reading frames ORFs) belongs to PB1-like viruses, SL2 (279,696 bp, 354 ORFs) to phiKZ-like viruses and SL4 (44,194 bp, 65 ORFs) to LUZ24-like viruses. Planktonic cells of four of five selected MDR-PA strains were suppressed by at least one phage with multiplicities of infection (MOIs) ranging from 1 to 10^−6^ for 16 h without apparent regrowth of bacterial populations. While SL2 was most potent in suppressing planktonic cultures the strongest anti-biofilm activity was observed with SL4. Phages were able to rescue bacteria-infected wax moth larvae (*Galleria melonella*) for 24 h, whereby highest survival rates (90%) were observed with SL1. Except for the biofilm experiments, the effect of a cocktail with all three phages was comparable to the action of the best phage alone; hence, there are no synergistic but also no antagonistic effects among phages. The use of a cocktail with these phages is therefore expedient for increasing host range and minimizing the development of phage resistance.

## 1. Introduction

Human phage therapy is currently being broadly reconsidered and multi-drug resistant organisms from the ESKAPE group (i.e., *Enterococcus faecium*, *Staphylococcus aureus*, *Klebsiella pneumoniae*, *Acinetobacter baumannii*, *Pseudomonas aeruginosa*, and *Enterobacter* species) are likely the prime candidates for which this treatment option might be realized in the Western world [1]. The interest in phage therapy is particularly strong for *P. aeruginosa*, as this species is a notorious source of bacteremia in burn victims, urinary-tract infections in catheterized patients, and hospital-acquired pneumonia and respiratory infections in cystic fibrosis patients [2]. Even worse, treatment becomes increasingly difficult, as *P. aeruginosa* is ecologically very flexible and possesses a gallery of different antibiotic-resistance mechanisms, e.g., different carbapenemases [3,4]. Principally, the enormous genomic plasticity of *P. aeruginosa* makes this species adaptable to numerous ecological niches and thus widespread in natural and domestic environments [5,6]. Consequently, the ubiquity and heterogeneity of *P. aeruginosa* has also led to the evolution of distinct phage species that can be globally isolated from a variety of many different environmental niches [7,8]. This is in part reflected by the fact that among members of ESKAPE the number of available phages from official phage collection centers is highest for *P. aeruginosa* [9]. Likewise, *Pseudomonas* phages also lead the list of completely sequenced genomes in GenBank {i.e., 152 *Pseudomonas* phages, versus 111 *Staphylococcus* phages, 29 *Klebsiella* phages, 27 *Acinetobacter* phages, 26 *Enterococcus* phages, and 10 *Enterobacter* phages (August 2017)}, which also reflects the scientific endeavor to find therapeutically useful phages. Despite the large inter-strain diversity of *P. aeruginosa* worldwide it appears that the genetic diversity of its viral predators seems relatively confined, since almost every new phage isolate shares >90% sequence identity at nucleotide level with known phages of the same genus [5,10]. However, even closely related phages may exhibit profound variations in phenotypic properties, such as different host ranges or different lysis efficiencies of their bacterial hosts [7,10]. Therefore, it is still worthwhile to isolate and characterize new *P. aeruginosa* phages to gain a more comprehensive appreciation of therapeutically useful or less useful phages. In our previous pilot study, we have isolated phages against ESKAPE from various aquatic environments in the vicinity of a hospital [9]. In this study, *P. aeruginosa* phages were obtained from 12 out of 16 different environmental sources (75%), while phages against the other members of ESKAPE were found in only 25 to 38% of samples [9]. Here, we describe the characterization of three selected phage isolates (each affiliated with a different phage genus, i.e., PB1-like, phiKZ-like and LUZ24-like viruses) using current clinically relevant multi-drug resistant *P. aeruginosa* strains as target hosts. 

## 2. Material and Methods

### 2.1. Bacterial Strains

In total, 24 multi-drug resistant *P. aeruginosa* strains (MDR-PA1-24) isolated from clinical specimen at the University Hospital RWTH Aachen, Germany, were used for determination of the host range of phages. Species identity was verified via MALDI-TOF mass spectrometry (Microflex LT, Bruker Daltonik GmbH, Bremen, Germany). For liquid and biofilm-infection assays five distinct MDR strains (MDR-PA1-5) were selected. Unless otherwise stated, bacterial strains were generally grown in Lysogeny Broth (LB) (NaCl 1% *w*/*v*, tryptone 1% *w*/*v*, and yeast extract 0.5% *w*/*v*). The automated antimicrobial susceptibility testing of clinical isolates was performed using the VITEK2 system (bioMérieux, Marcy-l’Étoile, France). The identification of carbapenem-resistance genes was performed by polymerase chain reaction (PCR) using the Xpert Carba-R kit (Cepheid, Sunnyvale, CA, USA) [11]. Bacterial strains were further differentiated via enterobacterial repetitive intergenic consensus PCR (ERIC-PCR) as described previously [12,13]. Briefly, ERIC-PCR was performed in a 50 µL volume and each reaction mixture contained 2.5 U of GoTaq G2 Flexi DNA Polymerase supplied with GoTaq Flexi Buffer (Promega, Madison, WI, USA), 4 mM MgCl_2_, 0.2 mM dNTPs (Roche Applied Science, Penzberg, Germany), 2.0 µM of each primer, and approximately 50 ng of template DNA. Genomic DNA from pure cultures was obtained using the QIAamp DNA Mini Kit (Qiagen, Hilden, Germany) according to the guidelines of the manufacturer. The concentration of each purified genomic DNA sample was measured with the NanoVue Plus (GE Healthcare, Little Chalfont, UK). The primers for ERIC-PCR were: “ERIC-1” 5′-ATGTAAGCTCCTGGGGATTCAC-3′ and “ERIC-2” 5′-AAGTAAGTGACTGGGGTGAGCG-3′. The thermal profile of the PCR reaction started with an initial denaturation at 95 °C for 7 min, followed by 35 cycles of denaturation at 94 °C for 30 s, primer annealing at 52 °C for 1 min and extension at 72 °C for 8 min, and one cycle of further extension at 72 °C for 16 min. The DNA-fragments generated via ERIC-PCR were run on a 1.5% TBE-agarose gel at 80 V for 3 h at room temperature and visualized using the GelStudio SA System (Analytik Jena, Jena, Germany). DNA fingerprint data were analyzed via GelQuest and ClusterVis (http://www.sequentix.de/gelquest/help/index.html). For calculation of distance matrices the Jaccard-index and for calculation of cluster trees the neighbor joining algorithm was used.

### 2.2. Phage Preparation and Storage

Initially, five distinct phages (designated as SL1–SL5), previously isolated from hospital sewage based on five different strains of MDR-PA as host [9], were used in this study. During the course of the experiments, phage SL3 could not consistently be grown on its host strain and was eventually disregarded for further experiments. In addition, genomic analysis revealed that phage SL5 is likely a temperate phage and thus was not further considered as a potential therapeutic agent. Therefore, the current study focuses on the three remaining phages: SL1 (host strain MDR-PA1), SL2 (host strain MDR-PA2), and SL4 (host strain MDR-PA4).

Phages were purified by successive single plaque isolation and propagation. In order to obtain high titer phage lysates phages were mixed with a mid-log-phase MDR-PA culture and shaken at 200 rpm overnight at 37 °C. After overnight incubation, the host–phage suspension was centrifuged at 2330× *g* for 10 min and filtered twice with a 0.45-µm-pore-size and a 0.2-µm-pore-size sterile filter. Phage titer was determined as the number of plaque forming units (PFU/mL) by the Double Agar Overlay Plaque Assay [14]. For short-term storage (e.g., up to two weeks), phage lysates were stored at 4 °C. For long-term storage phages were mixed with glycerol (20% (*v*/*v*)) in equal parts and stored in CryoPure Tubes (Sarstedt, Nuembrecht, Germany) at −196 °C in liquid nitrogen.

### 2.3. Burst Size Experiments

One-step growth curves (phage SL1 with strain MDR-PA1, phage SL2 with strain MDR-PA2, phage SL4 with strain MDR-PA4) were performed as described earlier [14] with modifications. Briefly, a bacterial culture (with an optical density at 590 nm of 0.4, equal to approximately 10^8^ cfu/mL) was mixed with a phage suspension (10^6^ pfu/mL) to obtain a multiplicity of infection of 0.01. Phages were allowed to adsorb for 10 min at 37 °C, after which the mixture was diluted to 10^−4^. Triplicate samples taken at 5 min intervals for 30 min and then at 10 min intervals for 90 min were mixed with bacterial cultures and plated in order to obtain countable plaques in the bacterial lawn after overnight incubation 37 °C. Phage titers obtained at begin and end of the experiments were used to estimate the burst size. 

### 2.4. Transmission Electron Microscopy (TEM)

High-titer phage lysates were transferred into HEPES buffer and allowed to adsorb on glow discharged formvar-carbon-coated nickel grids (Maxtaform, 200 mesh, Plano, Wetzlar, Germany) for 10 min. Samples on grids were stained by placing on a drop of 0.5% uranyl acetate or on 1% phosphotungstic acid (in aqua dest., Science Services GmbH, Munich, Germany). After air drying, samples were examined using a TEM LEO 906 (Carl Zeiss, Oberkochen, Germany), operating at an acceleration voltage of 60 kV. 

Wide-angle Dual Speed 2K-CCD-Camera 14 bit (Tröndle, TRS Moorenweis, Germany) and analysis software IMAGE SP Professional (SISPROG, Tröndle, Moorenweis, Germany) were used to photograph observations.

### 2.5. Phage Genome Sequencing and In Silico Genome Analysis

Whole genome sequencing was performed by use of the MiSeq platform (Illumina, San Diego, CA, USA) according to the product manual (Nextera XT DNA Sample Preparation Guide (ersion October 2012). Prior to the extraction of phage DNA, remnants of bacterial DNA were removed through a DNAse digest for 15 min. Phage genomic DNA extraction was performed using the QIAamp DNA Mini Kit (Qiagen, Hilden, Germany) according to the product manual (DNA Purification from Blood and Body Fluids Spin Protocol). For sequencing a paired-end library was generated using the Nextera XT Library Prep Kit and 2 × 150 bp reads were generated using the MiSeq v2 Reagent Kit. De novo assembly of reads was performed via the St. Petersburg genome assembler (SPAdes) [15]. Via genome wide BLASTn analysis, the most closely related phages deposited in GenBank were searched and the result visualized via BRIG [16]. Potential ORFs were identified using GeneMark.hmm [17] and annotation was performed using PHAST [18]. Translated ORFs were further compared with the five most closely related phages via BLASTx and then manually evaluated. Putative tRNA genes were identified using the tRNAscan-SE program [19]. The genome nucleotide sequences of the three phages SL1, SL2, and SL4 have been deposited to GenBank and are available under the accession-numbers: MF768470, MF805716, and MF768469.

### 2.6. Infection of Planktonic Cells

Infection of planktonic cells was done at the exponential phase of bacterial growth. To this end, an overnight pre-inoculum was used to inoculate 10 mL of fresh 2 × LB-medium to allow growth to approximately 5 × 10^8^ CFU mL^−1^. In all experiments, 100 µL of a host suspension were mixed with 100 µL of phages (at four different concentrations) to obtain four multiplicities of infection (MOIs) ranging from 1 to 10^−6^. Control experiments were performed with 2 × LB-medium mixed with an equal volume of phage buffer. The infection of planktonic cells was performed in 96-well microtiter plates. The microtiter plates were sealed with an adhesive tape, subsequently. With the aid of a sterile syringe, holes were made at the edge above every well to ensure ventilation and supply of oxygen. The microtiter plates were placed into the microplate reader SpectraMax i3 (Molecular Devices, Sunnyvale, CA, USA) and incubated for 16 h at 37 °C while horizontally shaking (system modus: moderate). The OD_590_ was measured at time intervals of 20 min for the entire duration of the experiment. All experiments were performed in triplicate. The OD_590_ data were analyzed by calculating the area under the curve using numerical integration with the formula $\sum_{i=0}^{48}f\left(i*\Delta t\right)*\Delta t$, with Δt=20 min, and f(i∗Δt) representing the OD_590_-values measured every 20 min for 16 h. 

To verify whether resistance against the phages had been evolved during the liquid infection assays, the content of selected wells of the microtiter plates was plated on solid medium. After overnight incubation, three to five colonies were pooled in liquid medium and again spread on agar plates with subsequent spotting of phages.

### 2.7. Infection of Biofilm-Associated Cells

Static biofilm assays were carried out in in 96-well microtiter plates as described previously [20] with the following modifications. An overnight pre-inoculum of bacteria was diluted to obtain a cellular suspension of 10^7^ CFU mL^−1^. One hundred microliters of this suspension (10^6^ CFU) was transferred into the well of the microtiter plate. Biofilms were allowed to form for 24 h or 48 h at 37 °C under aerobic and static conditions. After biofilm formation the wells were washed three times to remove all non-adherent bacteria. Phages (approximately 10^8^ pfu/well) were added to the air-dried biofilm for either 3 h or 24 h. In order to remove the phage solution and dissolved bacterial cells or cell remnants the wells were then washed three times. The biofilm was stained with 1% crystal violet (Merck, Darmstadt, Germany) for 15 min at room temperature. The cells were then washed with water, after which acetic acid (33% *v*/*v*) was added for 15 min to dissolve the stain. The eluted stain was transferred into another 96-well microtiter plate and its absorbance was read at an OD_590_ using the microplate reader SpectraMax i3 (Molecular Devices, Sunnyvale, CA, USA). All experiments were performed in triplicate. 

The above described biofilm experiments were independently repeated. However, instead of the crystal violet assay, which measures dead and living cells, biofilm reduction was assessed using the BacTitre Glo Microbial Cell Viability Assay Kit (Promega, Madison, WI, USA). With this approach the activity of living cells was determined via degradation of ATP by the enzyme luciferase leading to a luminescent signal proportional to the initial amount of the ATP [21]. Luminescence measurements were performed with microplate reader SpectraMax i3 (Molecular Devices, Sunnyvale, CA, USA). All experiments were performed in triplicate.

### 2.8. Galleria mellonella Larvae Model

The in vivo assay was conducted on a wax moth larvae model (*G. mellonella*, Biosystems Technology, Exeter, UK). Larvae were stored in the dark at 12 °C and were used within two weeks upon arrival. Experiments were performed in sterile petri-dishes (ten larvae per trial) and were conducted up to 72 h. Three forms of negative controls were used for each experiment: (i) uninfected larvae; (ii) larvae injected with 20 μL of 0.9% saline; and (iii) larvae receiving phage lysate. As positive control, larvae were injected with a lethal dose of bacteria. 

A dose of 1000 CFU/10µL of *P. aeruginosa* clinical isolates was found to be lethal for the larvae within 24 h and used for the subsequent experiments. Larvae were injected into the ventral side of their last pair of pseudopods with 10 μL of bacterial culture using a microtiter syringe (Hamilton, Company, Reno, NV, USA). After injection, larvae were incubated at 37 °C and examined at 24, 48 and 72 h post-infection by assessment of survival. Unless stated otherwise, the antibacterial activity of the three phages was assessed by injecting the infected larvae with 10 μL of phage lysate 1 h post-infection. For testing phage cocktails, phage lysates were mixed in equal amounts and applied 1 h post-infection. The survival rates of larvae (expressed as percentage) were read at 2, 24, 48 and 72 h post-infection. 

### 2.9. Phage Dosing and Statistical Analysis

In the above described infection assays, different, multiplicities of infection (MOI) were tested. Throughout the article, the term MOI is understood as “input-MOI”, considering that the actual number of phages reaching their target and successfully initiating an infection is unknown [22]. A Student’s *t*-test was performed to assess the significance of the antibacterial activity of phages in the liquid and biofilm infection assays. For assessing the significance of the protecting effect of phages for bacteria-infected larvae, the Fisher Exact Probability Test was performed.

## 3. Results

### 3.1. Host Range, Morphology and Growth Characteristics

**Figure 1:**
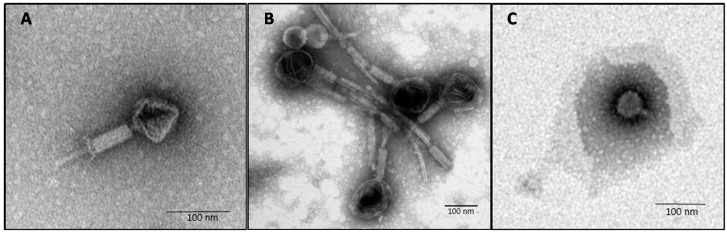
Electron micrograph images of phage SL1 (**A**); SL2 (**B**); and SL4 (**C**) infecting *P. aeruginosa*, negatively stained with: 0.5% uranyl acetate (**A**,**B**); or 1% phosphotungstic acid (**C**).

Local phages had been isolated from hospital sewage using multi-drug resistant bacterial hosts that were isolated from various acute and chronic sites of infection from the same hospital [9]. Based on different plaque morphology, three phages (SL1, SL2 and SL4) each obtained based on a different host were selected for further analysis. Besides individual antibiotic resistance profiles, the distinctiveness of 24 clinical isolates of multi-drug resistant *P. aeruginosa* (MDR-PA1–MDR-PA24) was verified based on genomic fingerprint analysis using ERIC-PCR (Supplementary Figure S1). Of these 24 MDR-PA isolates, the three phages were able to infect 10, 13, and 13 isolates (41.2%, 54.2% and 54.2%, respectively), based on clear lysis zones on agar plates. Taken together, 19 bacterial isolates were lysed by at least one phage (79%), leaving five isolates non-susceptible to the three phages. Based on transmission electron microscopy (TEM) phages SL1 and SL2 showed the typical morphology of *Myoviridae*, with an icosahedral head of approximately 70 nm and 120 nm in diameter, respectively (Figure 1A,B). 

The contractile tail including tail fibers was approximately 130 and 170 nm in length, respectively. Phage SL4 revealed the morphology of a *Podoviridae*, with an icosahedral head of about 55 nm and very short tail (about 20 nm) without visible tail fibers (Figure 1C). 

One-step growth experiments indicated the following latent period (LP) and average burst size (BS) per infected bacterial cell: SL1, 45 min (LP), 100 ± 11 phage particles (BS); SL2, 30 min (LP), 22 ± 6 phage particles (BS), and SL4, 50 min (LP), 16 ± 3 phage particles (BS).

### 3.2. Genome Analysis

Genome analysis revealed that phage SL1 belongs to the PB1-like viruses, SL2 is a “giant phage” belonging to the phiKZ-like viruses, and SL4 belongs to the LUZ24-like viruses. Major genomic features are summarized in Table 1. Further genome information is given in the Supplementary Material (i.e., Supplementary File S1, Supplementary Tables S1–S3, and Supplementary Figures S2–S4). 

**Figure 2:**
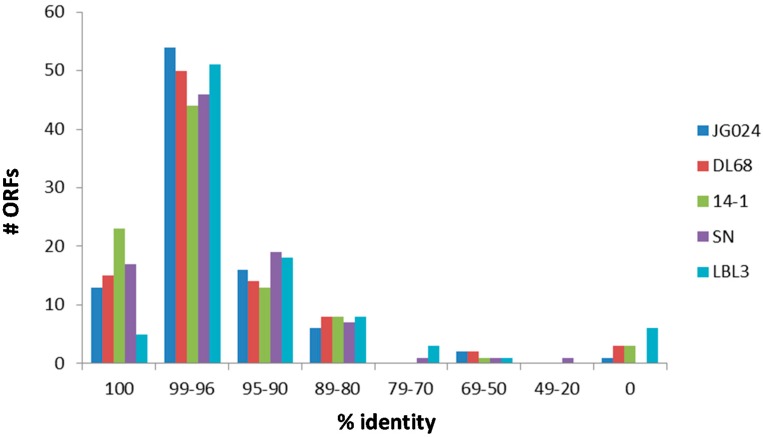
Sequence identity of translated ORFs of phage SL1 with five members of the PB1-like viruses (i.e., phages JG024, DL68, 14-1, SN, and LBL3).

When translating all ORFs into putative amino acid sequences, phage SL1 shares five to 23 ORFs with 100% sequence identity with the next related PB1-like viruses (Figure 2). However, the majority of ORFs of SL1 share 96–99% sequence identity at amino acid level with the closest relatives (Figure 2). Sequence identities of remaining ORFs decrease roughly consistently across the PB1-like phages. There are between one and six ORFs present in the genome of phage SL1 but missing in at least one of the other genomes of the phages shown in Figure 2.

One ORF of phage SL1 was 100% identical to a hypothetical protein of phage F8, and another ORF was 95% identical at amino acid level to a capsid and scaffold protein of phage PA5, which are both otherwise less closely related phages compared to those given in Figure 2.

**Figure 3:**
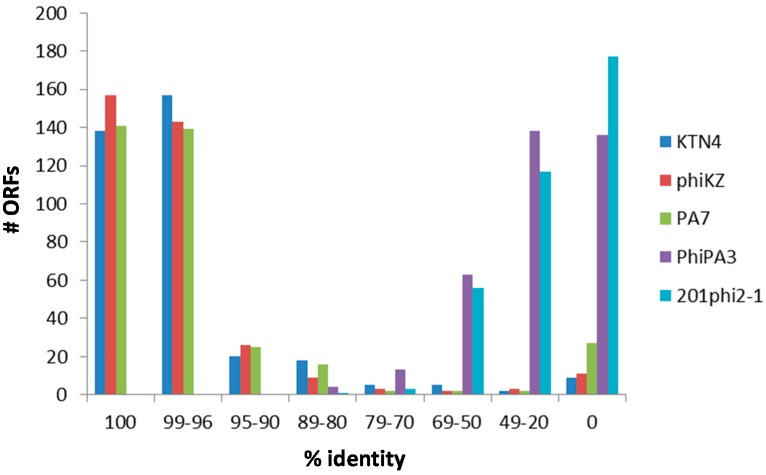
Sequence identity of translated ORFs of phage SL2 with five members of the phiKZ-like viruses (i.e., phages KTN4, phiKZ, PA7, PhiPA3, and 201phi2-1).

Overall, phage SL2 possesses between 138 and 157 ORFs (39–44%) with 100% identity at protein level to those detected in phages KTN4, phiKZ, and PA7 (Figure 3). A nearly equal number of ORFs shows sequence identities between 96% and 99%, whereas the sequence identities of the remaining ORFs decrease roughly consistently across those three phages. Furthermore, there are 9–27 ORFs present in phage SL2 but missing in at least one of the other three phages (Figure 3), all of which are hypothetical proteins. Six of these hypothetical proteins are only present in phage SL2 but not in the five phages depicted in Figure 3. This figure also shows that phages PhiPA3 and 201phi2-1 are clearly much more distantly related to phage SL2.

**Figure 4:**
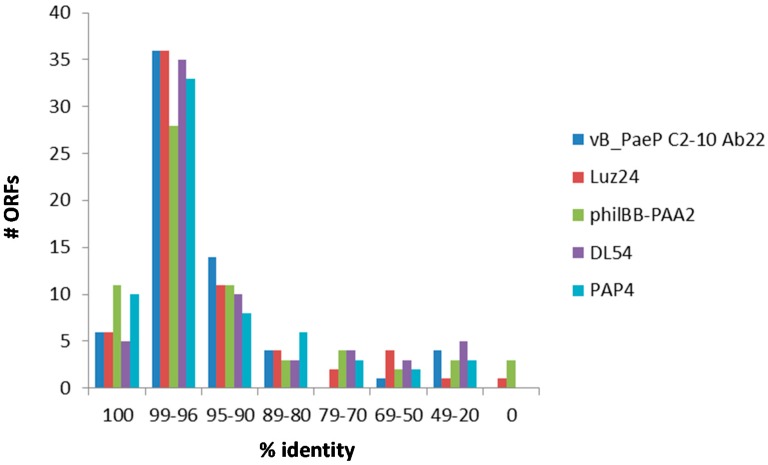
Sequence identity of translated ORFs of phage SL4 with five members of the LUZ24-like viruses (i.e., phages vB_PaeP C2-10 Ab22, Luz24, philBB-PAA2, DL54, and PAP4).

Phage SL4 possesses 4–11 ORFs with 100% identity at protein level with ORFs of those phages shown in Figure 4. The majority (28–36 ORFs) show sequence identities between 96% and 99%, followed by 8–14 ORFs with sequence identities between 90% and 95% at protein level (Figure 4).

Remaining ORFs, mostly hypothetical proteins, show even lower sequence identities. Furthermore, there are four ORFs in the genome of phage SL4 missing in at least one of the genomes of the five related phages (Figure 4). 

Lastly, genome analysis did not reveal the presence of virulence or lysogeny-associated proteins, principally qualifying the three phages SL1, SL2 and SL4 as safe biocontrol agents.

### 3.3. Liquid Infection Assays

**Figure 5:**
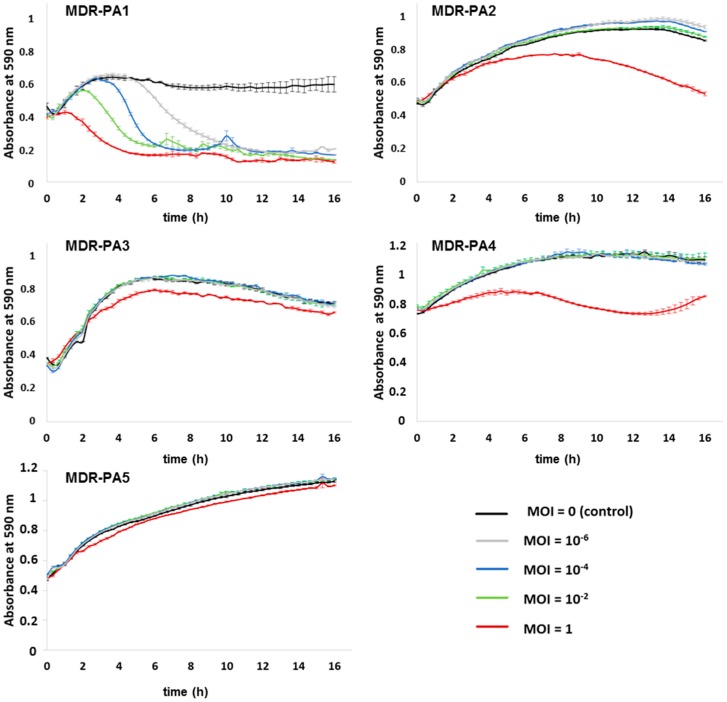
Activity of phage SL1 against planktonic cells of five different multi-drug resistant *P. aeruginosa* strains (MDR-PA1-5). Each experiment was performed in triplicate, and bars indicate standard deviation.

The efficacy of the three phages alone and as a cocktail was assessed by incubating the strains MDR-PA1-5 in liquid medium with single phages or in combination for 16 h using four different MOIs (i.e., MOI of 1, 10^−2^, 10^−4^, and 10^−6^, respectively). Phage SL1 was able to suppress the growth of its host strain MDR-PA1 at all MOIs, with the highest phage dilution initiating the decline after about 5 h of bacterial growth (Figure 5). 

**Figure 6:**
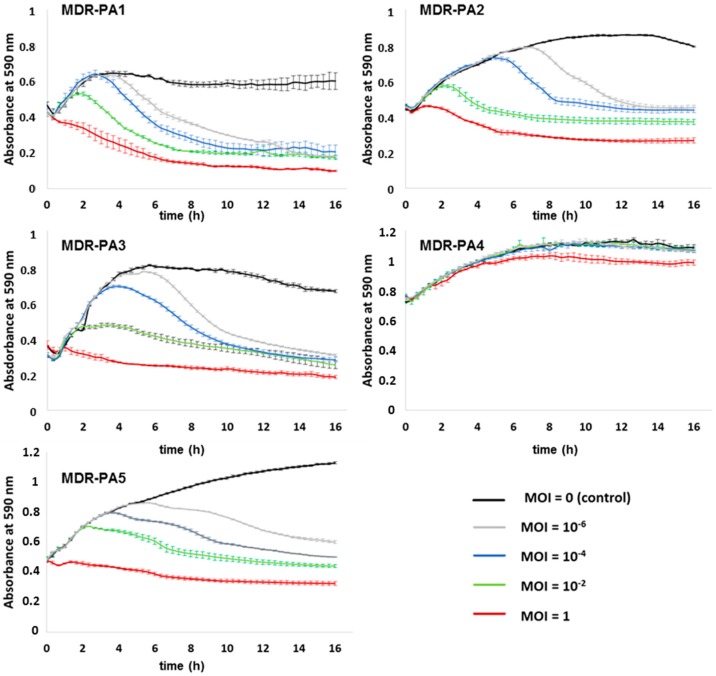
Activity of phage SL2 against planktonic cells of five different multi-drug resistant *P. aeruginosa* strains (MDR-PA1-5). Each experiment was performed in triplicate, and bars indicate standard deviation.

Bacterial populations remained suppressed for the whole time of the infection assay, without regrowth (Figure 5). For the remaining strains, SL1 had a moderate suppressive effect (MOI 1) except for MDR-PA5 (Figure 5). Phage SL2 suppressed four of the five MDR-PA strains efficiently at all MOIs including its host strain and there was no indication of regrowth for the period of 16 h (Figure 6). Strain MDR-PA4 was only marginally suppressed with the highest MOI of 1 (Figure 6).

**Figure 7:**
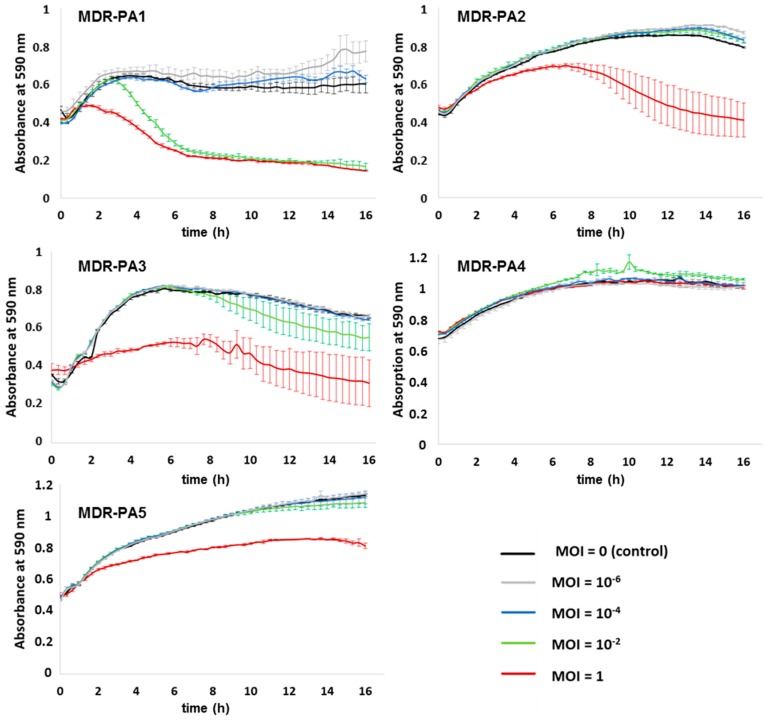
Activity of phage SL4 against planktonic cells of five different multi-drug resistant *P. aeruginosa* strains (MDR-PA1-5). Each experiment was performed in triplicate, and bars indicate standard deviation.

Phage SL4 suppressed MDR-PA1 at a MOI of 1 and 10^−2^, but higher phage dilutions had no suppressing effect on this bacterial strain (Figure 7). Strains MDR-PA2, MDR-PA3 and MDR-PA5 were suppressed by phage SL4 at MOI 1 (Figure 7), although not as efficiently as phage SL2 (Figure 6). Interestingly, SL4 did not suppress the growth of its host strain MDR-PA4 at all MOIs tested. 

**Figure 8:**
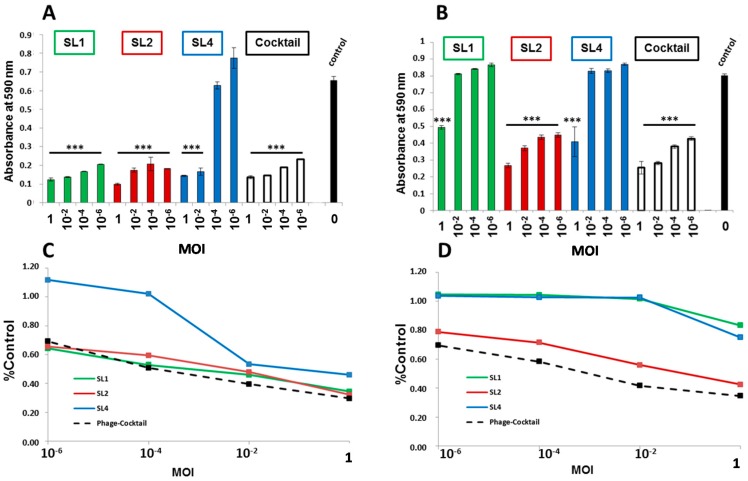
Reduction of multi-drug resistant *P. aeruginosa* strains: MDR-PA1 (**A**,**C**); and MDR-PA2 (**B**,**D**) in liquid medium after 16 h treatment with phages SL1, SL2, and SL4 in liquid culture using four different MOIs. (**A**,**B**) OD_590_-values after 16 h incubation with phage; and (**C**,**D**) area under the curves divided by the area under the curve of the control; Each experiment was performed in triplicate and the means ± standard errors are indicated. Statistical significance of bacterial reduction (compared to the control) was assessed by performing Student’s *t*-test. *p*-Values refer to the control: *** *p* < 0.001, ** *p* < 0.01, * *p* < 0.05.

A phage cocktail (equal mixture of all three phages) was tested on strains MDR-PA1 and MDR-PA2. The cocktail led to an efficient suppression at all MOIs tested, comparable to the outcome achieved with the best acting phage alone (Figure 8A,B). When taking into account the average suppressive effect over the entire period of the infection assay, which is expressed as the area under the absorbance curve, the phage cocktail was advantageous over the single phages at MOIs 10^−2^ and 1 in the case of MDR-PA1 and for all MOIs in the case of MDR-PA2 (Figure 8C,D). 

To verify whether resistance against the phages had been evolved, bacterial populations surviving the infection assay were encountered again with the initial phage or phage cocktail, using the spotting technique. All bacterial strains remained sensitive to at least one of the three phages. MDR-PA1 had developed resistance against SL1 and SL4 but not against SL2. MDR-PA2 remained sensitive against SL1 and SL2 but became resistant against SL4. MDR-PA3, which was only suppressed by SL2 and SL4, became only resistant against SL2. MDR-PA4 and MDR-PA5 remained sensitive against their only effective phages, namely SL1 and SL2, respectively. No development of resistance was observed during the timeframe of the experiment when phage cocktails were applied against strains MDR-PA1 and MDR-PA2. 

### 3.4. Biofilm Assays

**Figure 9:**
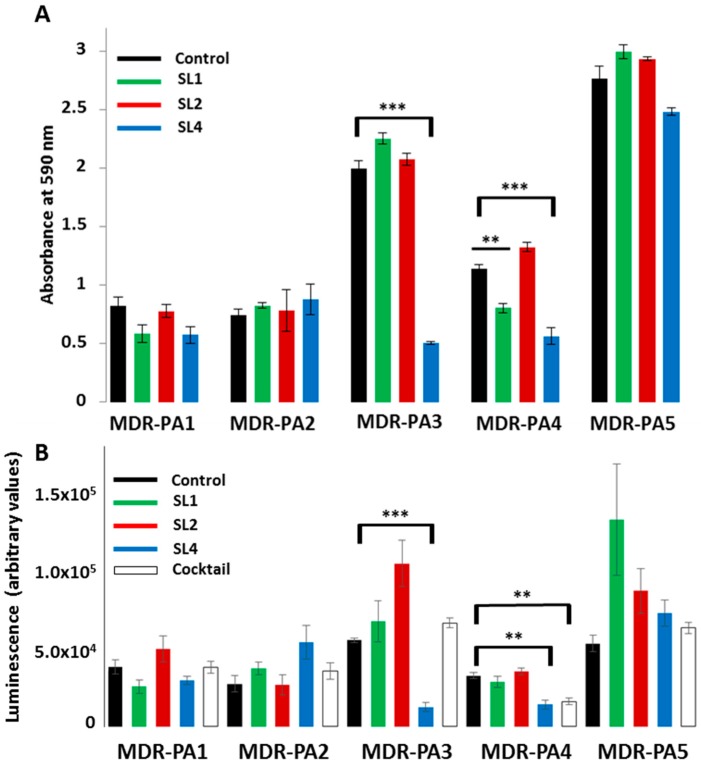
Reduction of 24 h-old biofilms of multi-drug resistant *P. aeruginosa* strains (MDR-PA1-5) by phages SL1, SL2, and SL4 and a cocktail with all three phages (phage incubation for 3 h, MOI 100): (**A**) Crystal violet assay; and (**B**) ATP-dependent luminescence assay. Each experiment was performed in triplicate and the means ± standard errors are indicated. Statistical significance of biofilm reduction was assessed by performing Student’s *t*-test. Note that the effect of the cocktail was only evaluated with the method in (**B**). *p*-Values: *** *p* < 0.001, ** *p* < 0.01, * *p* < 0.05.

Assessment of anti-biofilm activity against the five strains MDR-PA1-5 was performed in two different ways: first, by measuring the reduction of absorbance; and, second, by measuring reduction of bacterial activity via an ATP-based assay. With both measurements largely concordant anti-biofilm activity of phages were observed. Biofilm reduction through phages was possible for 24 h old biofilms but not for 48 h old biofilms. Application of phages to the biofilm was done for 3 h or for 24 h; however, the prolonged incubation time of phages did not lead to a stronger biofilm reduction. The biofilm of strain MDR-PA1 could tentatively be reduced by phages SL1 and SL4, however, with only marginal significance (Figure 9A,B). 

Biofilms of MDR-PA2 could not be reduced by any of the three phages. Conversely, the biofilm of MDR-PA3 and MDR-PA4 could be significantly reduced by SL4, supported by both evaluation assays (Figure 9A,B). In addition, the biofilm of MDR-PA4 could also be significantly reduced by phage SL1 (Figure 9A), which was not apparent with the ATP-dependent luminescence assay (Figure 9B). The strongest biofilm was formed by MDR-PA5 and could only be reduced by SL4, however, again, with only marginal significance. Phage SL2, which showed strongest potential in suppressing planktonic cells, was unable to reduce any biofilm of the five tested strains. Lastly, a phage cocktail with all three phages did not reveal anti-biofilm effects, except for strain MDR-PA4, whose activity, measured via the changes in ATP-levels, was significantly reduced by the cocktail (Figure 9B).

### 3.5. Wax Moth Assays

**Figure 10:**
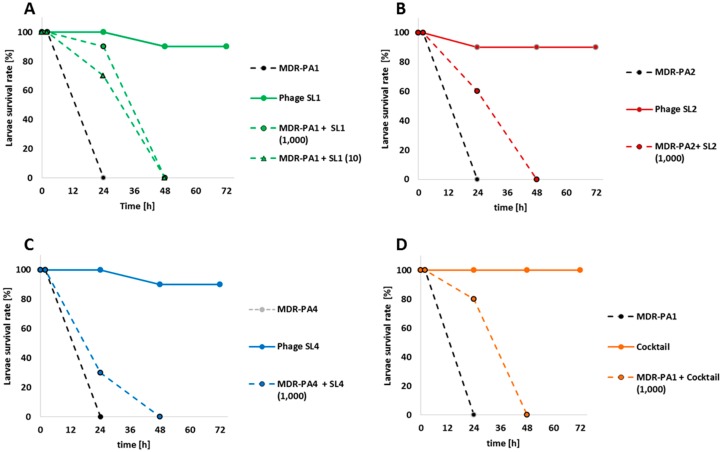
Percent Survival rate of larvae of Galleria mellonella after infection with multi-drug resistant *P. aeruginosa* strains (MDR-PA1, MDR-PA2 and MDR-PA4) and treatment with phages SL1 (**A**), SL2 (**B**), SL4 (**C**) or all phages together as a cocktail (**D**) 1 h post-infection. Numbers indicate MOI).

The therapeutic effect of the three phages was assessed against their host strains using the in vivo infection model of the wax moth *Galleria mellonella*. Control experiments, including sham-infected larvae with saline, and larvae receiving phage lysate only, led to a survival rate of larvae ranging from 90% to 100%. In addition, all larvae infected with bacteria but not treated with phages died within a period of 24 h (Figure 10). 

When applied 1 h post-infection with strain MDR-PA1, phage SL1 rescued 90% (MOI 1000; *p* < 0.001) and 70% (MOI 10; *p =* 0.003) of larvae for at least 24 h (Figure 10A). Phage SL2 achieved a 60% survival rate (*p =* 0.01) (Figure 10B), but phage SL4 only achieved a survival rate of 30% (Figure 10C). None of the bacteria-infected and phage treated larvae survived the period of 48 h. In order to achieve a prolonged survival of the larvae a phage cocktail against MDR-PA1 (MOI 1000) was applied. This cocktail rescued 80% of larvae for 24 h (*p* < 0.001) but again no larvae survived the period of 48 h (Figure 10D), which mirrors the outcome of the treatment with phage SL1 alone. A second application of phage SL1 after 24 h did not prolong the survival of the larvae as well. 

## 4. Discussion

Phages infecting *P. aeruginosa* are widespread in nature and there is an ongoing endeavor to explore their therapeutic potential. Based on genotypic and phenotypic features, *P. aeruginosa* phages from the order *Caudovirales* are classified into several distinct genera [23] and the phylogenetic inference based on the major capsid protein sequences groups these phages into at least ten clades that correspond well with the genus classification [24]. Studies evaluating the therapeutic potential of these phages have so far given some clades a clear preference, which is illustrated in a non-exhaustive compilation of studies in Supplemental Table S4. For instance, members of the PB1-like viruses have most frequently been explored as potential antibacterial agents (i.e., 19 out of a set of 34 selected studies, Supplemental Table S4). Next in line are members of the phiKMV-like viruses (i.e., these phages have been investigated in 11 out of 34 studies), whereas the other clades have been much less considered for therapeutic purposes (Supplemental Table S4). For instance, phiKZ-like viruses were tested in six and LUZ24-like viruses in only two out of 34 studies. Hence, besides testing a new isolate from the PB1-like viruses (SL1), our study provides some data on the antibacterial activity of two less frequently used clades (i.e., SL2 and SL4). 

Given the heterogeneity of *P. aeruginosa* populations the choice of bacterial strains for studying phages is a critical issue. Many studies have used the historical strain PAO1, which led to the most widely used model laboratory strain, Supplemental Table S4 [25,26]. Some studies relied exclusively on PAO1, while other studies focused on clinical isolates or other reference strains. Notably, eight out of 34 studies [27,28,29,30,31,32,33,34] used both clinical and reference strains (Supplemental Table S4). Clearly, reference strains allow comparison of phage performance across laboratories. However, it remains unclear how well information obtained from reference strains applies to topical clinical strains, which often reveal significant genetic and phenotypic differences [35,36] likely impacting also the infection efficiency of phages. Moreover, it seems that reference strain PAO1 also undergoes a continuing microevolution of geno- and phenotype which actually jeopardizes the reproducibility and comparability of research across worldwide laboratories [37]. Therefore, we decided in our study to use contemporary clinical multi-drug resistant *P. aeruginosa* isolates while bearing in mind that the “performance” of phages may differ when applied to other bacterial strains. 

The host spectrum of phage SL1 (41.2%) lies in between the range of other members of PB1-like viruses which varies between 17.2% and 67.2% depending on phage species [32]. The reported host range of phiKZ-like viruses varies between 23.9% and 46.5% depending on phage species [32], hence, with 53.4%, phage SL2 exhibits a slightly broader host range. Notably, in our study, phage SL4 infected half of the tested isolates which clearly exceeds the reported host range of phage LUZ24, with 22.4% [32]. However, the observed host range should be interpreted with caution. Since the host range in this study was determined by spotting of concentrated phage lysates, it cannot be ruled out that in some cases lysis occurred due to other effects, such as “lysis from without” [38] or presence of bacteriocins in the phage lysates. 

At genomic level phages SL1, SL2 and SL4 exhibit sequence identity levels with described phage species >90%, which is in keeping with the observation that new phage isolates rarely represent novel phylogenetic lineages [5,10]. Even though the major biological properties of closely related phage isolates may be largely comparable, it appears that each phage isolate shows genetic and phenotypic variations [7,10], which may affect host range and lysis efficiency. This distinctiveness of each phage isolate may be explained by the variations in the high number of ORFs with unknown functions [39]. Because of this large proportion of “viral dark matter” [40], the genomic comparison across phages as sole criterion allows no confident prediction of their therapeutic suitability, instead in vitro/in vivo infection assays are always necessary.

In our study phages decreased planktonic cells consistently for 16 h, albeit in a MOI dependent manner. Clearly the most rapid reduction was always achieved with the highest MOI, however, with phages SL1 and SL2 suppression of bacteria was possible even at the lowest MOI of 10^−6^. We are not aware of a study reporting a successful application of MOIs < 10^−4^ against MDR-PA strains; for instance, Henry et al. (2013) used a MOI of 10^−3^ [41], while otherwise typical MOIs used in liquid infection assays against *P. aeruginosa* range somewhere between 1 and 10^−2^ [28,42,43,44,45,46] or higher [47]. In light of the concerns that the patient’s immune system may eliminate or inactivate a substantial proportion of administered therapeutic phages before they enter the site of infection [48,49], an extant effectivity at very low MOIs is reassuring, as it still argues for the functioning of phage therapy as long as a few intact virions reach their target host. 

Phage SL1 controlled its host strain at all tested MOIs efficiently, but the other four clinical isolates were only marginally suppressed at the highest MOI of 1. In contrast, the giant phage SL2 reduced four of five MDR-PA strains at all MOIs tested. A different picture was seen with phage SL4, which exerted influence only until a MOI of 10^−2^ and, surprisingly, did not lyse its host strain in liquid medium. This was still true when the experiment was repeated with an elevated MOI of 10, data not shown. Apparently, phage SL4 lyses its host strain MDR-PA4 only at much higher MOIs (e.g., those typically achieved on agar plates, ranging somewhere between 100 and 1000 [38]. Considering the successful propagation of this phage from effluent using the same bacterial strain, we hypothesize the presence of some unknown phage-promoting factor(s) present in the effluent but absent in our laboratory tests. Another explanation could be the production of pigments, such as pyocyanin, as strain MDR-PA4 was the strongest pigment producer in our study. Pyocyanin can generate reactive oxygen species, such as H_2_O_2_ which is known to inactivate phages [50]. It is therefore possible that this molecule, or other reactive oxygen species, would have inhibited the phage in the liquid assay but not in the biofilm experiments where multiple washing steps occurred prior to phage application. This is a testable hypothesis that could be addressed in future studies.

In a number of liquid infection studies, it has been shown that the initial phage-driven decline of *P. aeruginosa* was followed by regrowth of bacterial populations, indicative of the evolvement of phage resistance [28,42,43,44,45,47,51,52]. This observation differs from our study, as regrowth after decline of bacteria did not occur within the timeframe of the experiment (except for phage SL1 and strain MDR-PA4, Figure 5). At the end of the 16 h infection assays, remaining bacterial cells were still viable on solid media but resistance against their initial predator was only observed in few cases. In eleven out of 15 tested phage-bacteria combinations (Figure 5, Figure 6 and Figure 7), a more or less strong suppression of bacteria was possible. Of these 11 cases, resistance had evolved only in four cases (confirmed twice independently with the MOIs of 1 and 10^−2^). In three of those four cases, regrowth had not occurred within the 16 h of the infection assay. We presume that that development of resistance either demanded a trade-off (loss) in growth fitness as described previously [53] or that resistance occurred at a very late stage such that it did not become apparent within the timeframe of the experiment. 

The use of phage cocktails instead of single phages is usually favored. Besides increasing the principle host range [54], a more efficient bacterial suppression can be achieved [53] and phage resistance may be hampered by multiple phages [42,55], however, not necessarily. For instance, the application of five different phages spanning three phage genera enabled a greater recovery of strain PAO1 (within a few hours) with a more generalized resistance mechanism than occurred with any of the five phages alone [44]. Conversely, Alves et al. (2016) [42] showed that a mixture of six phages (comprising four phage genera) enabled a full inhibition of PAO1 without regrowth. Since both studies used phages from three identical phage genera, one is tempted to speculate that the fourth phage genus applied by Alves et al. [42] a LUZ24-like virus, was crucial for avoidance of resistance. Thus far, LUZ24-like viruses have successfully been combined with PB1-like viruses (Supplemental Table S4) [42,56], and we are not aware of a study in which PB1-like and LUZ24-like viruses were tested in combination with phiKZ-like. Since members of these three genera alter the physiology of *P. aeruginosa* very differently during infection [5,57], they could either negatively or positively interfere with each other when applied in combination. Our data indicate no sign of negative interactions among the three phage genera as suppression of planktonic *P. aeruginosa* was also possible with phage cocktails. Although phages did not act synergistically (i.e., end concentrations of bacteria were not significant different from the action of the best phage alone), as a cocktail there was a more consistent suppression over time, which was particularly apparent for MDR-PA 2 (Figure 8D). This slight advantage of combined versus single phages could be important during phage therapy, as the bacterial pathogen is under more stress which would foster the possible synergy of phages with the patient’s immune system [58]. Hence, the use of a phage cocktail combining these three genera is recommendable. 

The elimination or reduction of the complex architecture of bacterial biofilm using phages is a greater challenge than the bacterial clearance of planktonic cells [59]. This was clearly evident in our study, since a significant reduction was only achieved with phage SL1 (one bacterial strain) and with phage SL4 (two bacterial strains), while phage SL2 failed to reduce the biofilm formed by the five MDR-PA isolates. One could speculate that phage size matters, since SL4 is smallest in our study (LUZ24-like virus) and SL2 a giant phage. However the close relative of SL2, namely phage KTN4 has proved to function well as anti-biofilm agent at least against the reference strain PAO1 [32]. Since *P. aeruginosa* biofilms can vary in their individual architecture [60] it is possible that the strain-specific biofilms in our study have hampered an improved anti-biofilm activity of the phages. This could probably be overcome by prolonged treatment periods combined with repeated dosing of phages as suggested previously [59]. Nonetheless, it should also be kept in mind that biofilms generated in the laboratory are not identical to in vivo biofilms [61]. In chronic infections, such as cystic fibrosis seeding of new aggregates by planktonic bacterial cells is a critical mechanism for the spreading, formation and persistence of biofilms [62]. Even if phages have limited utility in eliminating high-density biofilms, they may still be of therapeutic value by reducing the number of bacterial migrants required for new aggregate formation and dissemination of biofilms [29].

When applied as single agents, a significant proportion of larvae were rescued with phages SL1 and SL2, but not with SL4, although the latter was the more potent biofilm reducer. The fact that the phage cocktail tested against MDR-PA1 was as successful as the phage SL1 alone (Figure 10), again suggested that no competing interactions among the three phages occurred, and hence the combination of these three phages in a cocktail is possible. A limitation in our in vivo experiments was that no *P. aeruginosa*-infected larvae survived a period of 48 h despite phage treatment. One possibility could be the regrowth of phage-resistant bacterial clones inside the larvae. Another plausible explanation could be the relatively high bacterial load (i.e., 1000 CFU per larvae) required to achieve a lethal dose within 24 h. Although not explicitly tested in our study, phage-mediated killing may have led to the massive release of toxic compounds, e.g., endotoxins, and, consequently to the death of the larvae. For instance, a lethal dose of reference strain PAO1 for larvae was achieved already with 10 CFU [30,32] and, consequently, lower amounts of endotoxin must have been released upon phage treatment.

In conclusion, we have isolated and described three novel phages with close affiliation to phage species from three recognized genera. These phages suppress planktonic cells of MDR-PA clinical strains even at very low MOIs and development of resistance was not apparent within the timeframe of the experiments in most cases. The potential for biofilm reduction is given, however, modified phage applications are necessary to better exploit the anti-biofilm activity of phages, e.g., via repeated dosing. The *Galleria mellonella* infection model confirmed a protective effect of the phages, although the experimental time window was small, due to the burden of a high bacterial load. It is also possible that the observed effects of phages may imply to some extent the phenomenon of pseudolysogeny [63], which we did not further investigate in this study. Apart from additional in vivo experimentation, another big challenge for realizing phage therapy in future will be to shed more light into the “viral dark matter” hidden behind the high number of ORFs with unknown functions that still constitute the major part of the phage genomes. 

## Figures and Tables

**Table 1:** Nucleotide sequence identity of phages SL1, SL2, and SL4 with known phages of the same genus.

Novel Phages	Genome Size (bp)	# tRNAs	% GC	Related Phages	# ORFS	Accession Number	% Identity
**Phage SL1 (91 ORFs)**	65,849	0	55.6	JG024	93	GU815091.1	96
				14-1	90	FM897211.1	96
				DL68	92	KR054033.1	96
				SN	92	FM887021.1	96
				LBL3	88	FM201281.1	94
**Phage SL2 (355 ORFS)**	279,696	5	36.9	KTN4	368	KU521356.1	99
				phiKZ	369	AF399011.1	99
				PA7	337	JX233784.1	98
				PhiPA3	375	HQ630627.1	80
				201phi2-1	461	EU197055.1	69
**Phage SL4 (65 ORFs)**	44,194	2	52.3	philBB-PAA2	69	KF856712.1	95
				vB PaeP C2-10 Ab22	71	LN610578.1	94
				DL54	71	KR054029.1	93
				PaP4	70	KC294142.1	92
				LUZ24	68	AM910650.1	91

## Supplementary Materials

Supplementary information accompanies this paper at www.mdpi.com/1999-4915/9/11/315/s1. Supplementary file S1: description of genomic features of phages SL1, SL2, and SL4; Table S1: list of ORFs identified in the genome of phage SL1 using PHAST; Table S2: list of ORFs identified in the genome of phage SL2 using PHAST; Table S3: list of ORFs identified in the genome of phage SL4 using PHAST; Table S4: compilation of studies investigating the antibacterial activity of phages against P. aeruginosa; Figure S1: ERIC-PCR and resistance profiles of 24 *P. aeruginosa* isolates (MDR-PA1-24); Figure S2: visualisation of the BLASTn results for SL1 using BRIG; Figure S3: visualisation of the BLASTn results for SL2 using BRIG; Figure S4: visualisation of the BLASTn results for SL4 using BRIG.
