# Supplementary material for: Differential Effect of Newly Isolated Phages Belonging to PB1-Like, phiKZ-Like and LUZ24-Like Viruses against Multi-Drug Resistant Pseudomonas aeruginosa under Varying Growth Conditions
Source: Viruses. 2017 Oct 27;9(11):315. doi: 10.3390/v9110315 (PMC5707522; doi:10.3390/v9110315)
Supplement: Supplementary file 1 [file viruses-09-00315-s001.zip › SupplementalFiguresRevision.pdf]

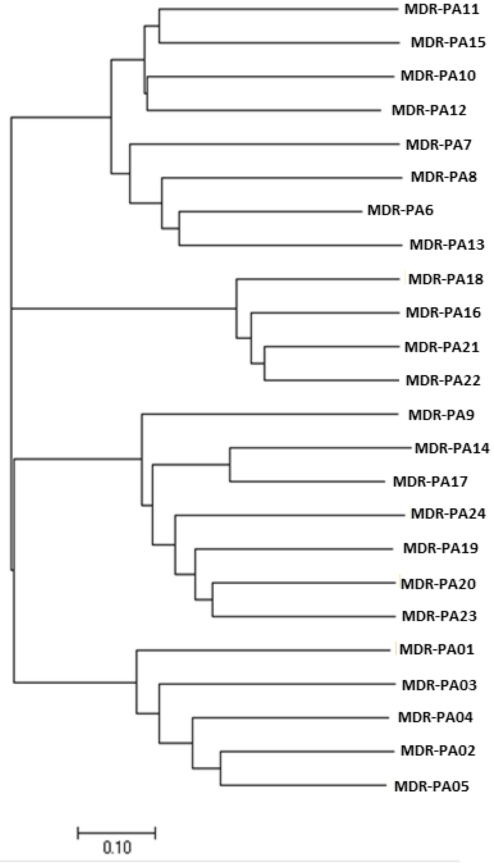

Isolate	Penicilline	Cephalosporine		Carbapeneme		Fluorchinolone	Aminogykoside			Polymyxine	Monobactam	Carbapenemase
	TZP	CAZ	FEP	IMP	MPM	CIP	GM	AK	TM	COL	AZN	
MDR-PA11	n.d.	n.d.	n.d.	n.d.	n.d.	n.d.	n.d.	n.d.	n.d.	n.d.	n.d.	n.d.
MDR-PA15	n.d.	n.d.	n.d.	n.d.	n.d.	n.d.	n.d.	n.d.	n.d.	n.d.	n.d.	n.d.
MDR-PA10	R	R	R	I	I	R	R	I	S	S	R	neg.
MDR-PA12	n.d.	n.d.	n.d.	n.d.	n.d.	n.d.	n.d.	n.d.	n.d.	n.d.	n.d.	n.d.
MDR-PA7	R	R	R	R	R	R	R	S	R	S	I	neg.
MDR-PA8	R	R	R	R	R	R	S	R	R	S	I	VIM
MDR-PA6	R	R	R	I	I	R	S	S	S	S	R	neg.
MDR-PA13	n.d.	n.d.	n.d.	n.d.	n.d.	n.d.	n.d.	n.d.	n.d.	n.d.	n.d.	n.d.
MDR-PA18	R	R	R	R	R	R	R	S	R	S	I	VIM
MDR-PA16	R	R	R	R	R	R	R	S	R	S	I	VIM
MDR-PA21	R	R	R	R	R	R	S	S	S	n.d.	I	neg.
MDR-PA22	R	R	R	R	R	R	S	S	S	S	R	neg.
MDR-PA9	R	R	R	R	R	R	R	R	S	n.d.	R	neg.
MDR-PA14	R	R	R	R	R	R	S	S	S	n.d.	n.d.	neg.
MDR-PA17	R	R	R	R	R	R	S	S	S	S	I	neg.
MDR-PA24	R	R	R	R	R	R	R	R	R	S	I	IMP1
MDR-PA19	R	R	R	R	I	R	S	S	S	S	R	neg.
MDR-PA20	R	R	R	R	R	R	S	S	S	S	R	neg.
MDR-PA23	R	R	R	R	R	R	R	S	R	S	R	VIM
MDR-PA01	R	R	R	R	I	R	R	S	S	S	I	neg.
MDR-PA03	R	R	R	R	I	I	S	S	S	S	R	OXA-48
MDR-PA04	R	R	R	R	R	R	n.d.	S	S	S	n.d.	Metallo
MDR-PA02	R	R	R	R	R	R	R	R	R	S	R	GES
MDR-PA05	R	R	R	R	R	R	S	S	S	S	R	neg.

Fig. S1 Neighbor-joining dendrogram of ERIC-PCR results for multi-drug resistant *P. aeruginosa* isolates (MDR-PA1-24) and resistance profiles. Piperacillin/Tazobactam (TZP), Ceftazidim (CAZ), Cefepim (FEP), Imipenem (IMP), Meropenem (MPM), Ciprofloxacin (CIP), Gentamicin (GM), Amikacin (AK), Tobramycin (TM), Colistin (COL) und Aztreonam (AZN). R= resistant, I= intermediate, S= sensitive, n.d.: not determined; neg = no known carbapnemases were detected via PCR;

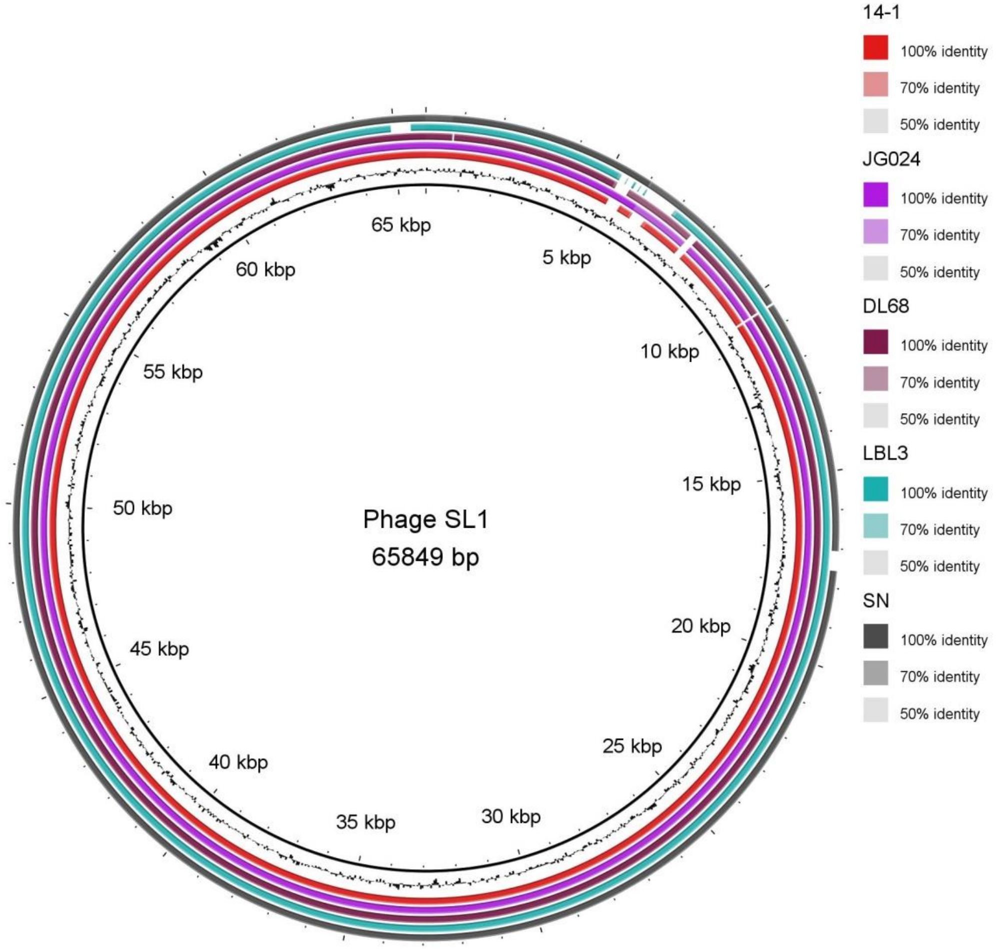

Fig. S2 Visualisation of the BLASTn results. Comparison of phage SL1 with five members of the PB1-like viruses

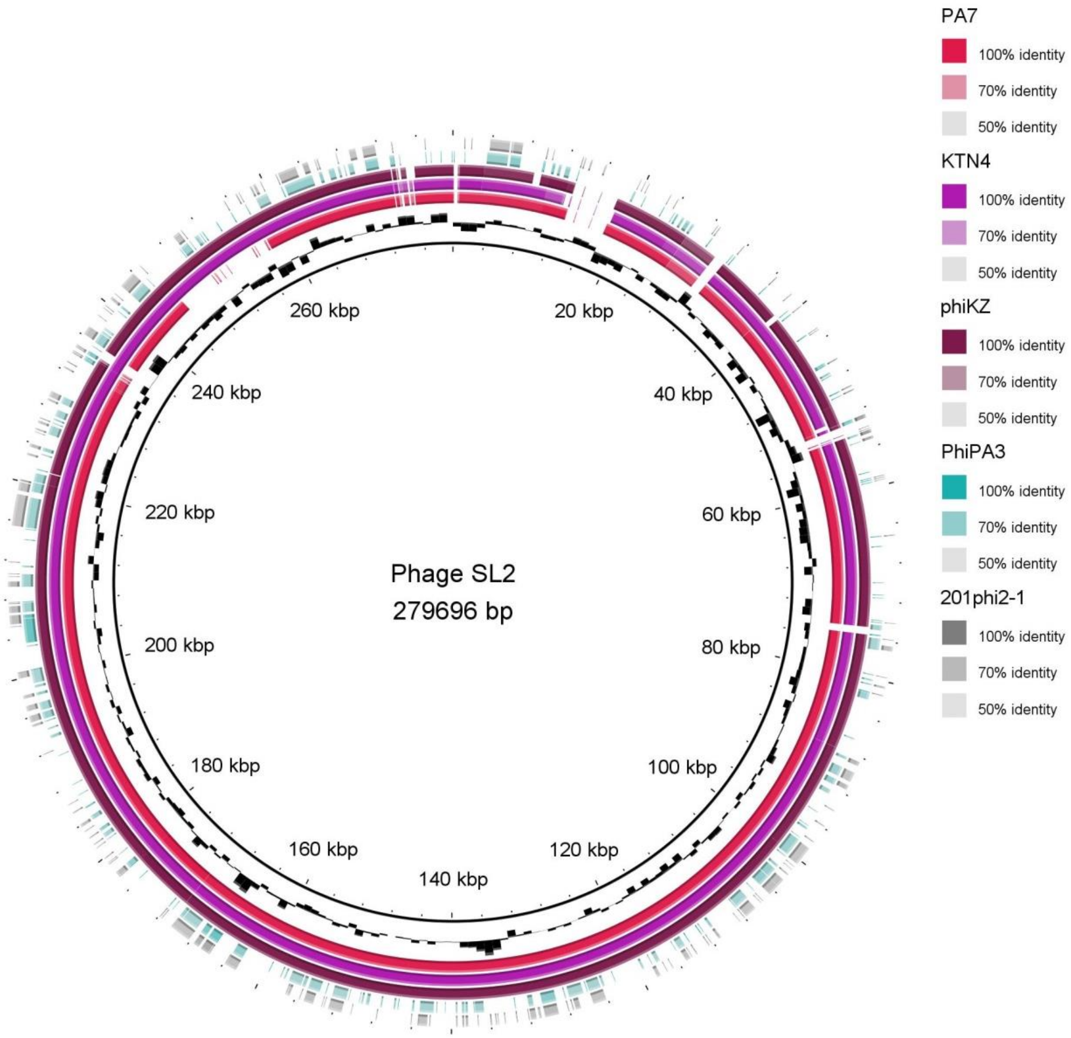

Fig. S3 Visualisation of the BLASTn results. Comparison of phage SL2 with five members of the phiKZ-like viruses

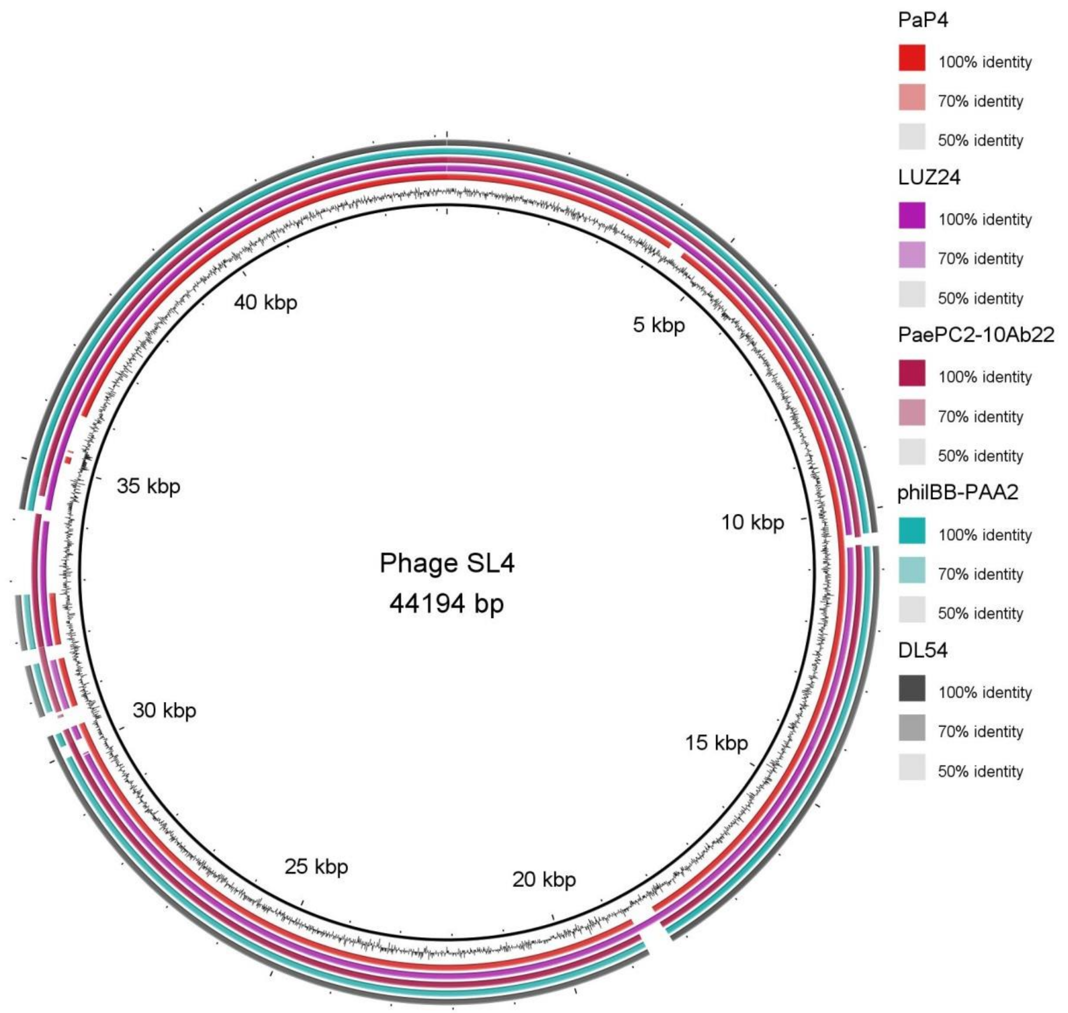

Fig. S4 Visualisation of the BLASTn results. Comparison of phage SL4 with five members of the LUZ24-like viruses
