# Supplementary material for: Differential Effect of Newly Isolated Phages Belonging to PB1-Like, phiKZ-Like and LUZ24-Like Viruses against Multi-Drug Resistant Pseudomonas aeruginosa under Varying Growth Conditions
Source: Viruses. 2017 Oct 27;9(11):315. doi: 10.3390/v9110315 (PMC5707522; doi:10.3390/v9110315)
Supplement: Supplementary file 1 [file viruses-09-00315-s001.zip › Table S1 ORFs SL1.docx]

|  | **Table S1 List of ORFs identified in the genome of phage SL1*** | |
| --- | --- | --- |
|  |  |  |
| 1 | 112..1011 | PHAGE_vB_PaeM_PAO1_Ab27_NC_026586: hypothetical protein; PP_00001; phage(gi764159610) |
| 2 | 1014..1451 | PHAGE_SN_NC_011756: hypothetical protein; PP_00002; phage(gi218457811) |
| 3 | 1538..2317 | PHAGE_SN_NC_011756: hypothetical protein; PP_00003; phage(gi218457810) |
| 4 | 2320..2718 | PHAGE_SN_NC_011756: hypothetical protein; PP_00004; phage(gi218457809) |
| 5 | 2763..3113 | PHAGE_SN_NC_011756: hypothetical protein; PP_00005; phage(gi218457808) |
| 6 | 3113..3331 | PHAGE_14_1_NC_011703: hypothetical protein; PP_00006; phage(gi218148545) |
| 7 | 3328..3711 | PHAGE_14_1_NC_011703: hypothetical protein; PP_00007; phage(gi218148544) |
| 8 | c(3747..5129) | PHAGE_SN_NC_011756: terminase, large subunit; PP_00008; phage(gi218457805) |
| 9 | 5305..5451 | PHAGE_SN_NC_011756: hypothetical protein; PP_00009; phage(gi218457804) |
| 10 | 5502..5675 | PHAGE_SN_NC_011756: hypothetical protein; PP_00010; phage(gi218457803) |
| 11 | 5730..6032 | PHAGE_SN_NC_011756: hypothetical protein; PP_00011; phage(gi218457802) |
| 12 | 6085..6771 | PHAGE_SN_NC_011756: hypothetical protein; PP_00012; phage(gi218457893) |
| 13 | 6774..6962 | PHAGE_SN_NC_011756: hypothetical protein; PP_00013; phage(gi218457892) |
| 14 | 7049..7300 | PHAGE_SN_NC_011756: hypothetical protein; PP_00014; phage(gi218457891) |
| 15 | 7297..7497 | PHAGE_JG024_NC_017674: hypothetical protein; PP_00015; phage(gi418486986) |
| 16 | 7494..7709 | PHAGE_PB1_NC_011810: hypothetical protein; PP_00016; phage(gi219523957) |
| 17 | 7706..7897 | PHAGE_SN_NC_011756: hypothetical protein; PP_00017; phage(gi218457888) |
| 18 | 7894..8106 | PHAGE_SN_NC_011756: hypothetical protein; PP_00018; phage(gi218457887) |
| 19 | 8134..8778 | PHAGE_SN_NC_011756: hypothetical protein; PP_00019; phage(gi218457886) |
| 20 | 8815..9144 | PHAGE_LBL3_NC_011165: hypothetical protein; PP_00020; phage(gi197261409) |
| 21 | 9206..9430 | PHAGE_SN_NC_011756: hypothetical protein; PP_00021; phage(gi218457884) |
| 22 | 9486..9707 | PHAGE_SN_NC_011756: hypothetical protein; PP_00022; phage(gi218457882) |
| 23 | 9760..10071 | PHAGE_SN_NC_011756: hypothetical protein; PP_00023; phage(gi218457881) |
| 24 | 10082..10705 | PHAGE_LMA2_NC_011166: hypothetical protein; PP_00024; phage(gi197261496) |
| 25 | 10896..11507 | PHAGE_14_1_NC_011703: hypothetical protein; PP_00025; phage(gi218148617) |
| 26 | c(11675..12244) | PHAGE_14_1_NC_011703: hypothetical protein; PP_00026; phage(gi218148616) |
| 27 | 12534..12707 | hypothetical; PP_00027 |
| 28 | c(13207..14946) | PHAGE_NH_4_NC_019451: putative primase; PP_00028; phage(gi418488522) |
| 29 | c(15094..15279) | PHAGE_SN_NC_011756: hypothetical protein; PP_00029; phage(gi218457876) |
| 30 | c(15285..16361) | PHAGE_14_1_NC_011703: hypothetical protein; PP_00030; phage(gi218148613) |
| 31 | c(16358..16807) | PHAGE_14_1_NC_011703: hypothetical protein; PP_00031; phage(gi218148612) |
| 32 | c(16831..17958) | PHAGE_SN_NC_011756: hypothetical protein; PP_00032; phage(gi218457873) |
| 33 | c(18145..18930) | PHAGE_SN_NC_011756: hypothetical protein; PP_00033; phage(gi218457872) |
| 34 | 19099..19521 | PHAGE_14_1_NC_011703: hypothetical protein; PP_00034; phage(gi218148609) |
| 35 | 19508..20695 | PHAGE_SN_NC_011756: putative ATP-dependent exonuclease V; PP_00035; phage(gi218457870) |
| 36 | 20857..21741 | PHAGE_SN_NC_011756: hypothetical protein; PP_00036; phage(gi218457869) |
| 37 | 21846..22847 | PHAGE_SN_NC_011756: hypothetical protein; PP_00037; phage(gi218457868) |
| 38 | 22936..23166 | PHAGE_SN_NC_011756: hypothetical protein; PP_00038; phage(gi218457867) |
| 39 | 23166..23387 | PHAGE_SN_NC_011756: hypothetical protein; PP_00039; phage(gi218457866) |
| 40 | 23371..23589 | PHAGE_SN_NC_011756: hypothetical protein; PP_00040; phage(gi218457865) |
| 41 | 23622..23855 | PHAGE_PB1_NC_011810: hypothetical protein; PP_00041; phage(gi219523933) |
| 42 | 23863..24069 | PHAGE_SN_NC_011756: hypothetical protein; PP_00042; phage(gi218457863) |
| 43 | 24069..24986 | PHAGE_DL60_NC_028745: thymidylate synthase thyX; PP_00043; phage(gi971482267) |
| 44 | 24988..25179 | PHAGE_SN_NC_011756: hypothetical protein; PP_00044; phage(gi218457861) |
| 45 | 25182..26210 | PHAGE_SN_NC_011756: hypothetical protein; PP_00045; phage(gi218457860) |
| 46 | 26286..26840 | PHAGE_KPP12_NC_019935: putative DNA polymerase; PP_00046; phage(gi431811137) |
| 47 | 26840..29947 | PHAGE_SN_NC_011756: DNA Polymerase III alpha subunit; PP_00047; phage(gi218457858) |
| 48 | 29937..30350 | PHAGE_SN_NC_011756: putative DNA helicase; PP_00048; phage(gi218457857) |
| 49 | 30347..31906 | PHAGE_SN_NC_011756: hypothetical protein; PP_00049; phage(gi218457856) |
| 50 | 31989..32621 | PHAGE_SN_NC_011756: hypothetical protein; PP_00050; phage(gi218457855) |
| 51 | 32710..33609 | PHAGE_DL68_NC_028971: hypothetical protein; PP_00051; phage(gi971761952) |
| 52 | 33663..34268 | PHAGE_SN_NC_011756: hypothetical protein; PP_00052; phage(gi218457853) |
| 53 | 34265..34819 | PHAGE_14_1_NC_011703: hypothetical protein; PP_00053; phage(gi218148590) |
| 54 | 34874..35785 | PHAGE_14_1_NC_011703: hypothetical protein; PP_00054; phage(gi218148589) |
| 55 | 36065..36316 | PHAGE_SN_NC_011756: hypothetical protein; PP_00055; phage(gi218457850) |
| 56 | c(36341..37003) | PHAGE_14_1_NC_011703: hypothetical protein; PP_00056; phage(gi218148587) |
| 57 | c(37003..37374) | PHAGE_SN_NC_011756: hypothetical protein; PP_00057; phage(gi218457848) |
| 58 | c(37433..40327) | PHAGE_DL68_NC_028971: tail fibers protein; PP_00058; phage(gi971761959) |
| 59 | c(40332..41846) | PHAGE_14_1_NC_011703: hypothetical protein; PP_00059; phage(gi218148584) |
| 60 | c(41843..43096) | PHAGE_SN_NC_011756: putative baseplate protein; PP_00060; phage(gi218457845) |
| 61 | c(43154..43819) | PHAGE_SN_NC_011756: putative baseplate protein; PP_00061; phage(gi218457844) |
| 62 | c(43875..44408) | PHAGE_SN_NC_011756: hypothetical protein; PP_00062; phage(gi218457843) |
| 63 | c(44408..45271) | PHAGE_SN_NC_011756: sructurall protein; PP_00063; phage(gi218457842) |
| 64 | c(45271..47847) | PHAGE_JG024_NC_017674: putative lytic tail protein; PP_00064; phage(gi418486935) |
| 65 | c(47851..48279) | PHAGE_SN_NC_011756: hypothetical protein; PP_00065; phage(gi218457840) |
| 66 | c(48289..48882) | PHAGE_SN_NC_011756: sructurall protein; PP_00066; phage(gi218457839) |
| 67 | c(48891..49295) | PHAGE_SN_NC_011756: sructurall protein; PP_00067; phage(gi218457838) |
| 68 | c(49430..49933) | PHAGE_SN_NC_011756: sructurall protein; PP_00068; phage(gi218457837) |
| 69 | c(49943..50374) | PHAGE_SN_NC_011756: hypothetical protein; PP_00069; phage(gi218457836) |
| 70 | c(50376..50726) | PHAGE_SN_NC_011756: sructurall protein; PP_00070; phage(gi218457835) |
| 71 | c(50723..51046) | PHAGE_SN_NC_011756: sructurall protein; PP_00071; phage(gi218457834) |
| 72 | c(51046..51498) | PHAGE_SN_NC_011756: sructurall protein; PP_00072; phage(gi218457833) |
| 73 | c(51557..53071) | PHAGE_KPP12_NC_019935: putative sructurall protein; PP_00073; phage(gi431811111) |
| 74 | c(53087..53668) | PHAGE_SN_NC_011756: hypothetical protein; PP_00074; phage(gi218457831) |
| 75 | c(53665..54216) | PHAGE_SN_NC_011756: sructurall protein; PP_00075; phage(gi218457830) |
| 76 | c(54224..54622) | PHAGE_SN_NC_011756: sructurall protein; PP_00076; phage(gi218457829) |
| 77 | c(54619..55086) | PHAGE_KPP12_NC_019935: putative sructurall protein; PP_00077; phage(gi431811107) |
| 78 | c(55101..55538) | PHAGE_SN_NC_011756: hypothetical protein; PP_00078; phage(gi218457827) |
| 79 | c(55640..56788) | PHAGE_DL68_NC_028971: capsid and scaffold protein; PP_00079; phage(gi971761980) |
| 80 | c(56798..57433) | PHAGE_SN_NC_011756: sructurall protein; PP_00080; phage(gi218457825) |
| 81 | c(57437..58870) | PHAGE_LMA2_NC_011166: putative sructurall protein; PP_00081; phage(gi197261440) |
| 82 | c(59383..59523) | PHAGE_LMA2_NC_011166: hypothetical protein; PP_00082; phage(gi197261439) |
| 83 | c(59520..59726) | PHAGE_LBL3_NC_011165: hypothetical protein; PP_00083; phage(gi197261346) |
| 84 | c(59745..60581) | PHAGE_SN_NC_011756: putative minor head protein; PP_00084; phage(gi218457821) |
| 85 | c(60581..62878) | PHAGE_SN_NC_011756: putative minor head protein; PP_00085; phage(gi218457820) |
| 86 | 63059..63460 | PHAGE_SN_NC_011756: hypothetical protein; PP_00086; phage(gi218457819) |
| 87 | 63492..63815 | PHAGE_SN_NC_011756: hypothetical protein; PP_00087; phage(gi218457818) |
| 88 | 63812..64015 | PHAGE_vB_PaeM_PAO1_Ab27_NC_026586: hypothetical protein; PP_00088; phage(gi764159613) |
| 89 | 64021..64332 | PHAGE_KPP12_NC_019935: hypothetical protein; PP_00089; phage(gi431811096) |
| 90 | 64581..64928 | PHAGE_14_1_NC_011703: hypothetical protein; PP_00090; phage(gi218148554) |
| 91 | 64948..65448 | PHAGE_SN_NC_011756: hypothetical protein; PP_00091; phage(gi218457815) |
|  | **c = complement*** |  |
|  |  |  |
|  | **Summary** | 64 hypothetical proteins |
|  |  | 13 structural proteins |
|  |  | 7 further specified structural proteins |
|  |  | 7 genes for host-independant DNA replication machinery |
|  |  | In total 91 ORFs |
