# Supplementary material for: Differential Effect of Newly Isolated Phages Belonging to PB1-Like, phiKZ-Like and LUZ24-Like Viruses against Multi-Drug Resistant Pseudomonas aeruginosa under Varying Growth Conditions
Source: Viruses. 2017 Oct 27;9(11):315. doi: 10.3390/v9110315 (PMC5707522; doi:10.3390/v9110315)
Supplement: Supplementary file 1 [file viruses-09-00315-s001.zip › Table S2 ORFs SL2.docx]

| **Table S2 List of ORFs identified in the genome of phage SL2*^1^** | |
| --- | --- |
|  |  |
| c(762..1166) | PHAGE_phiKZ_NC_004629: ORF005; PP_00003; phage(gi29134941) |
| c(1283..1804) | PHAGE_phiKZ_NC_004629: ORF004; PP_00004; phage(gi29134940) |
| c(1804..2310) | PHAGE_phiKZ_NC_004629: ORF003; PP_00005; phage(gi29134939) |
| c(2285..2869) | PHAGE_phiKZ_NC_004629: ORF002; PP_00006; phage(gi29134938) |
| c(2833..3222) | PHAGE_phiKZ_NC_004629: ORF001; PP_00007; phage(gi29134937) |
| 3567..5855 | PHAGE_phiKZ_NC_004629: ORF306; PP_00008; phage(gi29135242) |
| 5956..7119 | PHAGE_phiKZ_NC_004629: ORF305; PP_00009; phage(gi29135241) |
| 7119..7532 | PHAGE_phiKZ_NC_004629: ORF304; PP_00010; phage(gi29135240) |
| 7618..9510 | PHAGE_phiKZ_NC_004629: ORF303; PP_00011; phage(gi29135239) |
| 9736..10188 | PHAGE_phiKZ_NC_004629: ORF302; PP_00012; phage(gi29135238) |
| 10247..10331 | tRNA |
| 10343..10416 | tRNA |
| 10423..10498 | tRNA |
| 10583..11590 | PHAGE_phiKZ_NC_004629: ORF298; PP_00013; phage(gi29135234) |
| 11876..12388 | PHAGE_phiKZ_NC_004629: ORF297; PP_00014; phage(gi29135233) |
| 12550..12625 | tRNA |
| 12757..12942 | hypothetical; PP_00015 |
| 12935..13225 | PHAGE_phiKZ_NC_004629: ORF295; PP_00016; phage(gi29135231) |
| 13426..13584 | PHAGE_201phi2_1_NC_010821: hypothetical protein; PP_00017; phage(gi189490592) |
| 13574..14548 | PHAGE_201phi2_1_NC_010821: virion structural protein; PP_00018; phage(gi189490591) |
| 14545..16494 | PHAGE_201phi2_1_NC_010821: virion structural protein; PP_00019; phage(gi189490590) |
| 16491..17351 | PHAGE_201phi2_1_NC_010821: virion structural protein; PP_00020; phage(gi189490589) |
| 17351..18211 | PHAGE_201phi2_1_NC_010821: virion structural protein; PP_00021; phage(gi189490588) |
| 18371..18955 | PHAGE_phiKZ_NC_004629: ORF294; PP_00022; phage(gi29135230) |
| c(19058..19195) | hypothetical; PP_00023 |
| 19344..19420 | tRNA |
| 19550..19726 | hypothetical; PP_00024 |
| 19769..20179 | PHAGE_phiKZ_NC_004629: ORF293; PP_00025; phage(gi29135229) |
| 20260..20649 | PHAGE_phiKZ_NC_004629: ORF292; PP_00026; phage(gi29135228) |
| c(20712..21392) | PHAGE_phiKZ_NC_004629: ORF291; PP_00027; phage(gi29135227) |
| c(21393..21542) | hypothetical; PP_00028 |
| c(21561..22076) | PHAGE_phiKZ_NC_004629: ORF290; PP_00029; phage(gi29135226) |
| c(22082..22813) | PHAGE_phiKZ_NC_004629: ORF289; PP_00030; phage(gi29135225) |
| c(22980..23360) | PHAGE_phiKZ_NC_004629: ORF288; PP_00031; phage(gi29135224) |
| c(23503..23916) | PHAGE_PhiPA3_NC_028999: hypothetical protein; PP_00032; phage(gi971764429) |
| c(23919..24572) | PHAGE_phiKZ_NC_004629: ORF287; PP_00033; phage(gi29135223) |
| c(24721..24909) | hypothetical; PP_00034 |
| c(24933..26456) | PHAGE_phiKZ_NC_004629: ORF286; PP_00035; phage(gi29135222) |
| c(26514..27362) | PHAGE_phiKZ_NC_004629: ORF284; PP_00036; phage(gi29135220) |
| c(27404..27757) | hypothetical; PP_00037 |
| c(27822..28286) | PHAGE_phiKZ_NC_004629: ORF283; PP_00038; phage(gi29135219) |
| c(28302..28817) | PHAGE_phiKZ_NC_004629: ORF282; PP_00039; phage(gi29135218) |
| c(28918..29274) | PHAGE_phiKZ_NC_004629: ORF281; PP_00040; phage(gi29135217) |
| c(29315..29671) | PHAGE_phiKZ_NC_004629: ORF280; PP_00041; phage(gi29135216) |
| c(29685..30197) | PHAGE_phiKZ_NC_004629: ORF279; PP_00042; phage(gi29135215) |
| c(30230..30691) | PHAGE_PhiPA3_NC_028999: hypothetical protein; PP_00043; phage(gi971764415) |
| c(30691..31149) | hypothetical; PP_00044 |
| 31198..31353 | hypothetical; PP_00045 |
| c(31350..31787) | PHAGE_phiKZ_NC_004629: ORF278; PP_00046; phage(gi29135214) |
| c(31846..32145) | PHAGE_phiKZ_NC_004629: ORF277; PP_00047; phage(gi29135213) |
| c(32157..32678) | PHAGE_phiKZ_NC_004629: ORF276; PP_00048; phage(gi29135212) |
| c(32693..33052) | PHAGE_phiKZ_NC_004629: ORF275; PP_00049; phage(gi29135211) |
| c(33049..33288) | PHAGE_phiKZ_NC_004629: ORF274; PP_00050; phage(gi29135210) |
| c(33301..33987) | PHAGE_phiKZ_NC_004629: ORF273; PP_00051; phage(gi29135209) |
| c(34034..34369) | PHAGE_phiKZ_NC_004629: ORF272; PP_00052; phage(gi29135208) |
| c(34413..34778) | PHAGE_phiKZ_NC_004629: ORF271; PP_00053; phage(gi29135207) |
| c(34780..35157) | PHAGE_phiKZ_NC_004629: ORF270; PP_00054; phage(gi29135206) |
| c(35154..35366) | PHAGE_phiKZ_NC_004629: ORF269; PP_00055; phage(gi29135205) |
| c(35375..35677) | PHAGE_phiKZ_NC_004629: ORF268; PP_00056; phage(gi29135204) |
| c(35637..35789) | hypothetical; PP_00057 |
| c(35970..36272) | PHAGE_phiKZ_NC_004629: ORF267; PP_00058; phage(gi29135203) |
| c(36335..36766) | PHAGE_phiKZ_NC_004629: ORF266; PP_00059; phage(gi29135202) |
| c(36803..37240) | hypothetical; PP_00060 |
| c(37233..37529) | PHAGE_phiKZ_NC_004629: ORF265; PP_00061; phage(gi29135201) |
| c(37608..38027) | PHAGE_phiKZ_NC_004629: ORF264; PP_00062; phage(gi29135200) |
| c(38052..38330) | PHAGE_phiKZ_NC_004629: ORF263; PP_00063; phage(gi29135199) |
| c(38358..38591) | PHAGE_phiKZ_NC_004629: ORF262; PP_00064; phage(gi29135198) |
| c(38644..39066) | PHAGE_phiKZ_NC_004629: ORF261; PP_00065; phage(gi29135197) |
| c(39087..39617) | PHAGE_phiKZ_NC_004629: ORF208; PP_00066; phage(gi29135144) |
| c(39663..40022) | PHAGE_phiKZ_NC_004629: ORF260; PP_00067; phage(gi29135196) |
| c(40147..40581) | PHAGE_phiKZ_NC_004629: ORF259; PP_00068; phage(gi29135195) |
| c(40584..40958) | PHAGE_phiKZ_NC_004629: ORF258; PP_00069; phage(gi29135194) |
| c(41003..41689) | PHAGE_phiKZ_NC_004629: ORF257; PP_00070; phage(gi29135193) |
| c(41889..42344) | PHAGE_phiKZ_NC_004629: ORF255; PP_00071; phage(gi29135191) |
| c(42337..42711) | PHAGE_phiKZ_NC_004629: ORF254; PP_00072; phage(gi29135190) |
| c(43005..43244) | PHAGE_phiKZ_NC_004629: ORF253; PP_00073; phage(gi29135189) |
| c(43234..43611) | PHAGE_phiKZ_NC_004629: ORF252; PP_00074; phage(gi29135188) |
| c(43644..44099) | PHAGE_phiKZ_NC_004629: ORF251; PP_00075; phage(gi29135187) |
| c(44117..44461) | PHAGE_phiKZ_NC_004629: ORF250; PP_00076; phage(gi29135186) |
| c(44514..45308) | PHAGE_phiKZ_NC_004629: ORF249; PP_00077; phage(gi29135185) |
| c(45312..45671) | PHAGE_phiKZ_NC_004629: ORF248; PP_00078; phage(gi29135184) |
| c(45684..46091) | PHAGE_phiKZ_NC_004629: ORF247; PP_00079; phage(gi29135183) |
| c(46118..46267) | PHAGE_PhiPA3_NC_028999: hypothetical protein; PP_00080; phage(gi971764280) |
| c(46500..47156) | PHAGE_phiKZ_NC_004629: ORF246; PP_00081; phage(gi29135182) |
| c(47187..47621) | PHAGE_phiKZ_NC_004629: ORF245; PP_00082; phage(gi29135181) |
| c(47672..48028) | PHAGE_phiKZ_NC_004629: ORF244; PP_00083; phage(gi29135180) |
| c(48086..48982) | PHAGE_phiKZ_NC_004629: ORF243; PP_00084; phage(gi29135179) |
| c(49003..49440) | PHAGE_phiKZ_NC_004629: ORF242; PP_00085; phage(gi29135178) |
| c(49552..49881) | PHAGE_phiKZ_NC_004629: ORF241; PP_00086; phage(gi29135177) |
| c(50011..50493) | PHAGE_phiKZ_NC_004629: ORF240; PP_00087; phage(gi29135176) |
| c(50680..50802) | hypothetical; PP_00088 |
| c(50874..51167) | PHAGE_Lu11_NC_017972: hypothetical protein; PP_00089; phage(gi388684719) |
| c(51136..51267) | hypothetical; PP_00090 |
| c(51267..51767) | PHAGE_phiKZ_NC_004629: ORF238; PP_00091; phage(gi29135174) |
| c(51817..52650) | PHAGE_phiKZ_NC_004629: ORF237; PP_00092; phage(gi29135173) |
| c(52733..52975) | PHAGE_phiKZ_NC_004629: ORF235; PP_00093; phage(gi29135171) |
| c(53077..53886) | PHAGE_Acinet_Ac42_NC_014660: putative homing endonuclease GIY-YIG family; PP_00094; phage(gi311992724) |
| c(54124..55203) | PHAGE_phiKZ_NC_004629: ORF235; PP_00095; phage(gi29135171) |
| c(55203..55403) | hypothetical; PP_00096 |
| c(55431..55724) | PHAGE_phiKZ_NC_004629: ORF234; PP_00097; phage(gi29135170) |
| c(55830..56537) | PHAGE_phiKZ_NC_004629: ORF233; PP_00098; phage(gi29135169) |
| c(56546..56803) | hypothetical; PP_00099 |
| c(56800..57708) | PHAGE_phiKZ_NC_004629: ORF232; PP_00100; phage(gi29135168) |
| c(57731..58198) | PHAGE_phiKZ_NC_004629: ORF231; PP_00101; phage(gi29135167) |
| c(58198..58371) | hypothetical; PP_00102 |
| c(58473..58724) | hypothetical; PP_00103 |
| c(58756..59052) | PHAGE_phiKZ_NC_004629: ORF230; PP_00104; phage(gi29135166) |
| c(59109..59474) | PHAGE_phiKZ_NC_004629: ORF229; PP_00105; phage(gi29135165) |
| c(59531..60130) | PHAGE_phiKZ_NC_004629: ORF228; PP_00106; phage(gi29135164) |
| c(60143..60658) | PHAGE_phiKZ_NC_004629: ORF227; PP_00107; phage(gi29135163) |
| c(60667..60996) | PHAGE_phiKZ_NC_004629: ORF226; PP_00108; phage(gi29135162) |
| c(60983..61174) | PHAGE_phiKZ_NC_004629: ORF225; PP_00109; phage(gi29135161) |
| c(61297..61710) | PHAGE_phiKZ_NC_004629: ORF224; PP_00110; phage(gi29135160) |
| c(61718..62116) | PHAGE_phiKZ_NC_004629: ORF223; PP_00111; phage(gi29135159) |
| c(62305..62637) | PHAGE_phiKZ_NC_004629: ORF222; PP_00112; phage(gi29135158) |
| c(62707..63054) | PHAGE_phiKZ_NC_004629: ORF221; PP_00113; phage(gi29135157) |
| c(63157..63627) | PHAGE_phiKZ_NC_004629: ORF220; PP_00114; phage(gi29135156) |
| c(63624..64757) | PHAGE_phiKZ_NC_004629: ORF219; PP_00115; phage(gi29135155) |
| c(64807..65106) | hypothetical; PP_00116 |
| c(65217..65432) | PHAGE_phiKZ_NC_004629: ORF218; PP_00117; phage(gi29135154) |
| c(65452..65643) | PHAGE_phiKZ_NC_004629: ORF217; PP_00118; phage(gi29135153) |
| c(65701..66177) | PHAGE_phiKZ_NC_004629: ORF216; PP_00119; phage(gi29135152) |
| c(66268..66504) | PHAGE_phiKZ_NC_004629: ORF215; PP_00120; phage(gi29135151) |
| c(66507..67076) | PHAGE_phiKZ_NC_004629: ORF214; PP_00121; phage(gi29135150) |
| c(67100..67639) | PHAGE_phiKZ_NC_004629: ORF213; PP_00122; phage(gi29135149) |
| c(67653..67997) | PHAGE_phiKZ_NC_004629: ORF212; PP_00123; phage(gi29135148) |
| c(67994..68413) | PHAGE_phiKZ_NC_004629: ORF211; PP_00124; phage(gi29135147) |
| c(68426..68593) | hypothetical; PP_00125 |
| c(68809..68934) | hypothetical; PP_00126 |
| c(68946..69278) | PHAGE_phiKZ_NC_004629: ORF210; PP_00127; phage(gi29135146) |
| c(69406..70008) | PHAGE_phiKZ_NC_004629: ORF209; PP_00128; phage(gi29135145) |
| c(70116..71216) | PHAGE_phiKZ_NC_004629: ORF208; PP_00129; phage(gi29135144) |
| c(71320..71973) | PHAGE_phiKZ_NC_004629: ORF207; PP_00130; phage(gi29135143) |
| c(71973..72683) | PHAGE_phiKZ_NC_004629: ORF206; PP_00131; phage(gi29135142) |
| c(72926..73327) | PHAGE_phiKZ_NC_004629: ORF205; PP_00132; phage(gi29135141) |
| c(73344..73520) | hypothetical; PP_00133 |
| c(73492..73887) | PHAGE_phiKZ_NC_004629: ORF204; PP_00134; phage(gi29135140) |
| 74259..77168 | PHAGE_phiKZ_NC_004629: ORF203; PP_00135; phage(gi29135139) |
| 77180..77650 | PHAGE_phiKZ_NC_004629: ORF202; PP_00136; phage(gi29135138) |
| 77661..79610 | PHAGE_phiKZ_NC_004629: ORF201; PP_00137; phage(gi29135137) |
| c(79651..80199) | PHAGE_phiKZ_NC_004629: ORF200; PP_00138; phage(gi29135136) |
| c(80330..80761) | PHAGE_phiKZ_NC_004629: ORF199; PP_00139; phage(gi29135135) |
| c(80852..81340) | PHAGE_phiKZ_NC_004629: ORF198; PP_00140; phage(gi29135134) |
| c(81483..81845) | PHAGE_phiKZ_NC_004629: ORF197; PP_00141; phage(gi29135133) |
| c(81857..82219) | PHAGE_phiKZ_NC_004629: ORF196; PP_00142; phage(gi29135132) |
| c(82221..82595) | PHAGE_phiKZ_NC_004629: ORF195; PP_00143; phage(gi29135131) |
| c(82745..83101) | PHAGE_phiKZ_NC_004629: ORF194; PP_00144; phage(gi29135130) |
| c(83114..83476) | PHAGE_phiKZ_NC_004629: ORF193; PP_00145; phage(gi29135129) |
| c(83476..84201) | PHAGE_phiKZ_NC_004629: ORF192; PP_00146; phage(gi29135128) |
| c(84194..84985) | PHAGE_phiKZ_NC_004629: ORF191; PP_00147; phage(gi29135127) |
| c(85013..85453) | PHAGE_phiKZ_NC_004629: ORF190; PP_00148; phage(gi29135126) |
| c(85523..85774) | PHAGE_phiKZ_NC_004629: ORF189; PP_00149; phage(gi29135125) |
| c(85812..86870) | PHAGE_phiKZ_NC_004629: ORF188; PP_00150; phage(gi29135124) |
| c(86873..87202) | PHAGE_phiKZ_NC_004629: ORF187; PP_00151; phage(gi29135123) |
| c(87323..87727) | PHAGE_phiKZ_NC_004629: ORF186; PP_00152; phage(gi29135122) |
| c(87758..88000) | hypothetical; PP_00153 |
| c(88002..88496) | PHAGE_phiKZ_NC_004629: ORF185; PP_00154; phage(gi29135121) |
| c(88605..89297) | PHAGE_phiKZ_NC_004629: ORF184; PP_00155; phage(gi29135120) |
| c(89305..89610) | PHAGE_phiKZ_NC_004629: ORF183; PP_00156; phage(gi29135119) |
| c(89669..91663) | PHAGE_phiKZ_NC_004629: ORF182; PP_00157; phage(gi29135118) |
| c(91719..98432) | PHAGE_phiKZ_NC_004629: ORF181; PP_00158; phage(gi29135117) |
| 98508..100160 | PHAGE_phiKZ_NC_004629: ORF180; PP_00159; phage(gi29135116) |
| 100163..104518 | PHAGE_phiKZ_NC_004629: ORF178; PP_00160; phage(gi29135114) |
| 104502..104699 | PHAGE_201phi2_1_NC_010821: hypothetical protein; PP_00161; phage(gi189490426) |
| 104711..106270 | PHAGE_phiKZ_NC_004629: ORF177; PP_00162; phage(gi29135113) |
| 106424..107146 | PHAGE_phiKZ_NC_004629: ORF176; PP_00163; phage(gi29135112) |
| 107158..107970 | PHAGE_phiKZ_NC_004629: ORF175; PP_00164; phage(gi29135111) |
| c(108020..109084) | PHAGE_phiKZ_NC_004629: ORF174; PP_00165; phage(gi29135110) |
| c(109167..110093) | PHAGE_phiKZ_NC_004629: ORF173; PP_00166; phage(gi29135109) |
| c(110098..110538) | PHAGE_phiKZ_NC_004629: ORF172; PP_00167; phage(gi29135108) |
| c(110531..111292) | PHAGE_phiKZ_NC_004629: ORF171; PP_00168; phage(gi29135107) |
| c(111363..111887) | PHAGE_phiKZ_NC_004629: ORF170; PP_00169; phage(gi29135106) |
| 111967..112482 | PHAGE_phiKZ_NC_004629: ORF169; PP_00170; phage(gi29135105) |
| c(112528..113184) | PHAGE_phiKZ_NC_004629: ORF168; PP_00171; phage(gi29135104) |
| c(113234..113662) | PHAGE_phiKZ_NC_004629: ORF167; PP_00172; phage(gi29135103) |
| c(113681..113887) | hypothetical; PP_00173 |
| c(114030..114425) | PHAGE_phiKZ_NC_004629: ORF166; PP_00174; phage(gi29135102) |
| 114517..114693 | PHAGE_PhiPA3_NC_028999: hypothetical protein; PP_00175; phage(gi971764393) |
| c(114736..117228) | PHAGE_phiKZ_NC_004629: ORF165; PP_00176; phage(gi29135101) |
| 117293..118174 | PHAGE_phiKZ_NC_004629: ORF164; PP_00177; phage(gi29135100) |
| 118184..118450 | PHAGE_201phi2_1_NC_010821: hypothetical protein; PP_00178; phage(gi189490402) |
| c(118490..119677) | PHAGE_phiKZ_NC_004629: ORF163; PP_00179; phage(gi29135099) |
| c(119687..121255) | PHAGE_phiKZ_NC_004629: ORF162; PP_00180; phage(gi29135098) |
| c(121291..121926) | PHAGE_phiKZ_NC_004629: ORF161; PP_00181; phage(gi29135097) |
| c(122025..122498) | PHAGE_phiKZ_NC_004629: ORF160; PP_00182; phage(gi29135096) |
| c(122502..122906) | PHAGE_phiKZ_NC_004629: ORF159; PP_00183; phage(gi29135095) |
| c(122903..123853) | PHAGE_phiKZ_NC_004629: ORF158; PP_00184; phage(gi29135094) |
| c(123909..125246) | PHAGE_phiKZ_NC_004629: ORF157; PP_00185; phage(gi29135093) |
| 125581..125715 | hypothetical; PP_00186 |
| 125736..126137 | PHAGE_phiKZ_NC_004629: ORF156; PP_00187; phage(gi29135092) |
| 126193..127641 | PHAGE_phiKZ_NC_004629: ORF155; PP_00188; phage(gi29135091) |
| 127901..128071 | PHAGE_phiKZ_NC_004629: ORF154; PP_00189; phage(gi29135090) |
| c(128118..129032) | PHAGE_phiKZ_NC_004629: ORF153; PP_00190; phage(gi29135089) |
| 129129..130568 | PHAGE_phiKZ_NC_004629: ORF152; PP_00191; phage(gi29135088) |
| 130645..130995 | PHAGE_phiKZ_NC_004629: ORF151; PP_00192; phage(gi29135087) |
| 131019..131339 | PHAGE_phiKZ_NC_004629: ORF150; PP_00193; phage(gi29135086) |
| 131372..132046 | PHAGE_phiKZ_NC_004629: ORF149; PP_00194; phage(gi29135085) |
| 132049..132555 | PHAGE_phiKZ_NC_004629: ORF148; PP_00195; phage(gi29135084) |
| 132545..133261 | PHAGE_phiKZ_NC_004629: ORF147; PP_00196; phage(gi29135083) |
| c(133395..136676) | PHAGE_phiKZ_NC_004629: ORF146; PP_00197; phage(gi29135082) |
| c(136676..139045) | PHAGE_phiKZ_NC_004629: ORF145; PP_00198; phage(gi29135081) |
| c(139251..140033) | PHAGE_phiKZ_NC_004629: ORF144; PP_00199; phage(gi29135080) |
| c(140095..140628) | PHAGE_phiKZ_NC_004629: ORF143; PP_00200; phage(gi29135079) |
| c(140711..141106) | PHAGE_phiKZ_NC_004629: ORF142; PP_00201; phage(gi29135078) |
| c(141081..141326) | hypothetical; PP_00202 |
| c(141328..141834) | PHAGE_phiKZ_NC_004629: ORF141; PP_00203; phage(gi29135077) |
| c(141850..142413) | PHAGE_phiKZ_NC_004629: ORF140; PP_00204; phage(gi29135076) |
| c(142416..143312) | PHAGE_phiKZ_NC_004629: ORF139; PP_00205; phage(gi29135075) |
| c(143408..143896) | PHAGE_phiKZ_NC_004629: ORF138; PP_00206; phage(gi29135074) |
| c(143905..144387) | PHAGE_phiKZ_NC_004629: ORF137; PP_00207; phage(gi29135073) |
| c(144338..144940) | PHAGE_phiKZ_NC_004629: ORF136; PP_00208; phage(gi29135072) |
| c(144940..146331) | PHAGE_phiKZ_NC_004629: ORF135; PP_00209; phage(gi29135071) |
| c(146331..147704) | PHAGE_phiKZ_NC_004629: ORF134; PP_00210; phage(gi29135070) |
| c(147716..149104) | PHAGE_phiKZ_NC_004629: ORF133; PP_00211; phage(gi29135069) |
| c(149179..149511) | PHAGE_phiKZ_NC_004629: ORF132; PP_00212; phage(gi29135068) |
| c(149524..151839) | PHAGE_phiKZ_NC_004629: ORF131; PP_00213; phage(gi29135067) |
| 151937..153220 | PHAGE_phiKZ_NC_004629: ORF130; PP_00214; phage(gi29135066) |
| c(153271..155952) | PHAGE_phiKZ_NC_004629: ORF129; PP_00215; phage(gi29135065) |
| 155988..158162 | PHAGE_phiKZ_NC_004629: ORF128; PP_00216; phage(gi29135064) |
| 158173..159045 | PHAGE_phiKZ_NC_004629: ORF127; PP_00217; phage(gi29135063) |
| 159054..159497 | PHAGE_phiKZ_NC_004629: ORF126; PP_00218; phage(gi29135062) |
| c(159562..160194) | PHAGE_phiKZ_NC_004629: ORF125; PP_00219; phage(gi29135061) |
| c(160275..162440) | PHAGE_phiKZ_NC_004629: ORF124; PP_00220; phage(gi29135060) |
| c(162476..164107) | PHAGE_phiKZ_NC_004629: ORF123; PP_00221; phage(gi29135059) |
| c(164117..165199) | PHAGE_phiKZ_NC_004629: ORF122; PP_00222; phage(gi29135058) |
| c(165156..165608) | PHAGE_phiKZ_NC_004629: ORF121; PP_00223; phage(gi29135057) |
| c(165805..168048) | PHAGE_phiKZ_NC_004629: ORF120; PP_00224; phage(gi29135056) |
| c(168121..168654) | PHAGE_phiKZ_NC_004629: ORF119; PP_00225; phage(gi29135055) |
| 168794..170341 | PHAGE_phiKZ_NC_004629: ORF118; PP_00226; phage(gi29135054) |
| 170396..170764 | PHAGE_PhiPA3_NC_028999: hypothetical protein; PP_00227; phage(gi971764208) |
| c(171449..171754) | PHAGE_phiKZ_NC_004629: ORF117; PP_00228; phage(gi29135053) |
| c(171921..172196) | PHAGE_phiKZ_NC_004629: ORF116; PP_00229; phage(gi29135052) |
| c(172198..172341) | hypothetical; PP_00230 |
| c(172381..172593) | PHAGE_PhiPA3_NC_028999: hypothetical protein; PP_00231; phage(gi971764114) |
| c(172590..172745) | hypothetical; PP_00232 |
| c(172748..172975) | PHAGE_phiKZ_NC_004629: ORF115; PP_00233; phage(gi29135051) |
| c(173039..173323) | PHAGE_phiKZ_NC_004629: ORF114; PP_00234; phage(gi29135050) |
| c(173331..173594) | PHAGE_phiKZ_NC_004629: ORF113; PP_00235; phage(gi29135049) |
| c(173640..173915) | PHAGE_phiKZ_NC_004629: ORF112; PP_00236; phage(gi29135048) |
| c(173957..174454) | PHAGE_phiKZ_NC_004629: ORF111; PP_00237; phage(gi29135047) |
| c(174490..174873) | PHAGE_phiKZ_NC_004629: ORF110; PP_00238; phage(gi29135046) |
| c(174909..175160) | PHAGE_phiKZ_NC_004629: ORF112; PP_00239; phage(gi29135048) |
| c(175232..175654) | PHAGE_phiKZ_NC_004629: ORF109; PP_00240; phage(gi29135045) |
| c(175665..176021) | PHAGE_phiKZ_NC_004629: ORF108; PP_00241; phage(gi29135044) |
| c(176109..176549) | hypothetical; PP_00242 |
| c(176539..176856) | PHAGE_phiKZ_NC_004629: ORF107; PP_00243; phage(gi29135043) |
| c(176902..177063) | hypothetical; PP_00244 |
| c(177172..177510) | PHAGE_phiKZ_NC_004629: ORF106; PP_00245; phage(gi29135042) |
| c(177634..178122) | PHAGE_phiKZ_NC_004629: ORF104; PP_00246; phage(gi29135040) |
| c(178203..178595) | PHAGE_phiKZ_NC_004629: ORF103; PP_00247; phage(gi29135039) |
| c(178604..178729) | hypothetical; PP_00248 |
| c(178769..179191) | PHAGE_phiKZ_NC_004629: ORF102; PP_00249; phage(gi29135038) |
| c(179216..180598) | PHAGE_phiKZ_NC_004629: ORF101; PP_00049; phage(gi29135037) |
| c(180600..181181) | PHAGE_phiKZ_NC_004629: ORF100; PP_00050; phage(gi29135036) |
| c(181186..182595) | PHAGE_phiKZ_NC_004629: ORF099; PP_00051; phage(gi29135035) |
| c(182595..184223) | PHAGE_phiKZ_NC_004629: ORF098; PP_00052; phage(gi29135034) |
| c(184307..186757) | PHAGE_phiKZ_NC_004629: ORF097; PP_00053; phage(gi29135033) |
| c(186857..187972) | PHAGE_phiKZ_NC_004629: ORF096; PP_00054; phage(gi29135032) |
| c(188034..189677) | PHAGE_phiKZ_NC_004629: ORF095; PP_00055; phage(gi29135031) |
| c(189765..191282) | PHAGE_phiKZ_NC_004629: ORF094; PP_00056; phage(gi29135030) |
| c(191353..192651) | PHAGE_phiKZ_NC_004629: ORF093; PP_00057; phage(gi29135029) |
| c(192657..193979) | PHAGE_phiKZ_NC_004629: ORF092; PP_00058; phage(gi29135028) |
| c(193989..194519) | PHAGE_phiKZ_NC_004629: ORF091; PP_00059; phage(gi29135027) |
| c(194534..195463) | PHAGE_phiKZ_NC_004629: ORF090; PP_00060; phage(gi29135026) |
| c(195474..196637) | PHAGE_phiKZ_NC_004629: ORF089; PP_00061; phage(gi29135025) |
| 196677..197726 | PHAGE_phiKZ_NC_004629: ORF088; PP_00062; phage(gi29135024) |
| 197730..200645 | PHAGE_phiKZ_NC_004629: ORF087; PP_00063; phage(gi29135023) |
| c(200687..201955) | PHAGE_phiKZ_NC_004629: ORF086; PP_00064; phage(gi29135022) |
| c(201966..202415) | PHAGE_phiKZ_NC_004629: ORF085; PP_00065; phage(gi29135021) |
| c(202427..203668) | PHAGE_phiKZ_NC_004629: ORF084; PP_00066; phage(gi29135020) |
| 203770..205284 | PHAGE_phiKZ_NC_004629: ORF083; PP_00067; phage(gi29135019) |
| c(205388..205504) | PHAGE_PhiPA3_NC_028999: hypothetical protein; PP_00068; phage(gi971764162) |
| c(205756..207672) | PHAGE_phiKZ_NC_004629: ORF082; PP_00069; phage(gi29135018) |
| c(207774..209195) | PHAGE_phiKZ_NC_004629: ORF081; PP_00070; phage(gi29135017) |
| c(209258..210607) | PHAGE_phiKZ_NC_004629: ORF080; PP_00071; phage(gi29135016) |
| c(210594..211451) | PHAGE_phiKZ_NC_004629: ORF079; PP_00072; phage(gi29135015) |
| 211623..212117 | PHAGE_phiKZ_NC_004629: ORF078; PP_00073; phage(gi29135014) |
| 212129..213730 | PHAGE_phiKZ_NC_004629: ORF077; PP_00074; phage(gi29135013) |
| 214054..214191 | hypothetical; PP_00075 |
| c(214242..214385) | hypothetical; PP_00076 |
| c(215378..216916) | PHAGE_phiKZ_NC_004629: ORF075; PP_00077; phage(gi29135011) |
| c(216975..219008) | PHAGE_phiKZ_NC_004629: ORF074; PP_00078; phage(gi29135010) |
| c(219039..219941) | PHAGE_phiKZ_NC_004629: ORF073; PP_00079; phage(gi29135009) |
| c(220264..221448) | PHAGE_phiKZ_NC_004629: ORF071; PP_00080; phage(gi29135007) |
| c(221441..221554) | hypothetical; PP_00081 |
| 221625..221987 | PHAGE_201phi2_1_NC_010821: hypothetical protein; PP_00082; phage(gi189490285) |
| c(222029..223408) | PHAGE_phiKZ_NC_004629: ORF070; PP_00083; phage(gi29135006) |
| c(223377..225050) | PHAGE_phiKZ_NC_004629: ORF069; PP_00084; phage(gi29135005) |
| c(225067..226632) | PHAGE_phiKZ_NC_004629: ORF068; PP_00085; phage(gi29135004) |
| c(226758..227498) | PHAGE_phiKZ_NC_004629: ORF067; PP_00086; phage(gi29135003) |
| c(227586..228416) | PHAGE_phiKZ_NC_004629: ORF066; PP_00087; phage(gi29135002) |
| c(228429..229505) | PHAGE_phiKZ_NC_004629: ORF065; PP_00088; phage(gi29135001) |
| c(229558..230121) | PHAGE_phiKZ_NC_004629: ORF064; PP_00089; phage(gi29135000) |
| c(230141..230698) | PHAGE_phiKZ_NC_004629: ORF063; PP_00090; phage(gi29134999) |
| c(230699..231109) | PHAGE_phiKZ_NC_004629: ORF062; PP_00091; phage(gi29134998) |
| c(231203..231667) | PHAGE_phiKZ_NC_004629: ORF061; PP_00092; phage(gi29134997) |
| c(231654..231989) | PHAGE_phiKZ_NC_004629: ORF060; PP_00093; phage(gi29134996) |
| c(232041..232652) | PHAGE_phiKZ_NC_004629: ORF059; PP_00094; phage(gi29134995) |
| c(232722..232904) | hypothetical; PP_00095 |
| c(233027..233452) | PHAGE_phiKZ_NC_004629: ORF058; PP_00096; phage(gi29134994) |
| c(233465..233848) | PHAGE_phiKZ_NC_004629: ORF057; PP_00097; phage(gi29134993) |
| c(233897..234082) | PHAGE_PhiPA3_NC_028999: hypothetical protein; PP_00098; phage(gi971764135) |
| c(235065..235886) | PHAGE_phiKZ_NC_004629: ORF056; PP_00099; phage(gi29134992) |
| c(235949..237088) | PHAGE_phiKZ_NC_004629: ORF055; PP_00100; phage(gi29134991) |
| c(237482..239362) | PHAGE_phiKZ_NC_004629: ORF054; PP_00101; phage(gi29134990) |
| c(239675..240040) | PHAGE_phiKZ_NC_004629: ORF053; PP_00102; phage(gi29134989) |
| 240122..241213 | PHAGE_phiKZ_NC_004629: ORF052; PP_00103; phage(gi29134988) |
| c(241257..241976) | PHAGE_phiKZ_NC_004629: ORF051; PP_00104; phage(gi29134987) |
| c(241963..242139) | hypothetical; PP_00105 |
| c(242238..244433) | PHAGE_phiKZ_NC_004629: ORF050; PP_00106; phage(gi29134986) |
| c(244634..245050) | PHAGE_phiKZ_NC_004629: ORF049; PP_00107; phage(gi29134985) |
| c(245085..245468) | PHAGE_phiKZ_NC_004629: ORF048; PP_00108; phage(gi29134984) |
| c(245541..246077) | PHAGE_phiKZ_NC_004629: ORF047; PP_00109; phage(gi29134983) |
| c(246074..246508) | PHAGE_phiKZ_NC_004629: ORF046; PP_00110; phage(gi29134982) |
| c(246781..247116) | hypothetical; PP_00111 |
| c(247157..247546) | PHAGE_phiKZ_NC_004629: ORF044; PP_00112; phage(gi29134980) |
| c(247597..248670) | PHAGE_phiKZ_NC_004629: ORF043; PP_00113; phage(gi29134979) |
| c(248684..249097) | hypothetical; PP_00114 |
| 249249..250049 | PHAGE_phiKZ_NC_004629: ORF042; PP_00115; phage(gi29134978) |
| c(250158..250385) | hypothetical; PP_00116 |
| c(250453..250740) | hypothetical; PP_00117 |
| c(250719..251036) | hypothetical; PP_00118 |
| c(251048..251521) | PHAGE_phiKZ_NC_004629: ORF041; PP_00119; phage(gi29134977) |
| c(251561..252043) | PHAGE_phiKZ_NC_004629: ORF040; PP_00120; phage(gi29134976) |
| c(252098..253078) | PHAGE_phiKZ_NC_004629: ORF039; PP_00121; phage(gi29134975) |
| c(253132..253644) | PHAGE_phiKZ_NC_004629: ORF038; PP_00122; phage(gi29134974) |
| c(253892..254713) | PHAGE_phiKZ_NC_004629: ORF037; PP_00123; phage(gi29134973) |
| c(254749..255561) | PHAGE_phiKZ_NC_004629: ORF036; PP_00124; phage(gi29134972) |
| c(255665..256480) | PHAGE_phiKZ_NC_004629: ORF035; PP_00125; phage(gi29134971) |
| c(256490..257260) | PHAGE_phiKZ_NC_004629: ORF034; PP_00126; phage(gi29134970) |
| c(257356..257559) | hypothetical; PP_00127 |
| c(257567..258436) | PHAGE_phiKZ_NC_004629: ORF033; PP_00128; phage(gi29134969) |
| 258527..259765 | PHAGE_phiKZ_NC_004629: ORF032; PP_00129; phage(gi29134968) |
| c(259812..260174) | hypothetical; PP_00130 |
| c(260165..261361) | PHAGE_phiKZ_NC_004629: ORF031; PP_00131; phage(gi29134967) |
| c(261489..261671) | hypothetical; PP_00132 |
| c(261671..262552) | PHAGE_phiKZ_NC_004629: ORF030; PP_00133; phage(gi29134966) |
| c(262584..264671) | PHAGE_phiKZ_NC_004629: ORF029; PP_00134; phage(gi29134965) |
| 264800..265762 | PHAGE_phiKZ_NC_004629: ORF028; PP_00135; phage(gi29134964) |
| 265779..268475 | PHAGE_phiKZ_NC_004629: ORF027; PP_00136; phage(gi29134963) |
| 268472..270130 | PHAGE_phiKZ_NC_004629: ORF026; PP_00137; phage(gi29134962) |
| 270270..272423 | PHAGE_phiKZ_NC_004629: ORF025; PP_00138; phage(gi29134961) |
| c(272477..272743) | PHAGE_phiKZ_NC_004629: ORF024; PP_00139; phage(gi29134960) |
| c(273154..273489) | PHAGE_phiKZ_NC_004629: ORF021; PP_00140; phage(gi29134957) |
| c(273793..274059) | hypothetical; PP_00141 |
| c(274063..274626) | PHAGE_phiKZ_NC_004629: ORF022; PP_00142; phage(gi29134958) |
| c(274848..275183) | hypothetical; PP_00143 |
| 275491..275604 | hypothetical; PP_00144 |
| c(275898..276227) | PHAGE_phiKZ_NC_004629: ORF018; PP_00145; phage(gi29134954) |
| c(276290..276682) | PHAGE_phiKZ_NC_004629: ORF017; PP_00146; phage(gi29134953) |
| c(276711..276854) | hypothetical; PP_00147 |
| c(276911..277207) | PHAGE_phiKZ_NC_004629: ORF016; PP_00148; phage(gi29134952) |
| c(277403..278404) | PHAGE_phiKZ_NC_004629: ORF015; PP_00149; phage(gi29134951) |
| c(278510..279622) | PHAGE_phiKZ_NC_004629: ORF014; PP_00150; phage(gi29134950) |
| ***c = complement** |  |
|  |  |
| **Summary** | 344 hypothetical Proteins (97%) |
|  | 5 tRNAs |
|  | 4 Structural proteins |
|  | 1 homing endonuclease GIY-YIG family; PP_00094; phage(gi311992724) |
|  | In total 354 ORFs |

^1^ Results have been obtained via PHAST-analysis. More detailed genome information was obtained by comparing the deduced protein sequences of the 354 ORFs with the genome of phage KTN4 from which a structural proteome analysis (ESI-MS/MS) had previously led to the identification of 111 virion-associated gene products (Danis-Wlodarczyk et al. 2016). Phage SL2 shares a sequence identity of 98-100% at protein level with those annotated gene products of KTN4 except for the Thymidylate synthase gene (77% sequence identity at amino acid level).

Reference:

Danis-Wlodarczyk, K.; Vandenheuvel, D.; Jang, H. B.; Briers, Y.; Olszak, T.; Arabski, M.; Wasik, S.; Drabik, M.; Higgins, G.; Tyrrell, J.; Harvey, B. J.; Noben, J.-P.; Lavigne, R.; Drulis-Kawa, Z. A proposed integrated approach for the preclinical evaluation of phage therapy in Pseudomonas infections. Sci. Rep. 2016, 6, doi:10.1038/srep28115.
