# Supplementary material for: Differential Effect of Newly Isolated Phages Belonging to PB1-Like, phiKZ-Like and LUZ24-Like Viruses against Multi-Drug Resistant Pseudomonas aeruginosa under Varying Growth Conditions
Source: Viruses. 2017 Oct 27;9(11):315. doi: 10.3390/v9110315 (PMC5707522; doi:10.3390/v9110315)
Supplement: Supplementary file 1 [file viruses-09-00315-s001.zip › Table S3 ORFs SL4.docx]

|  | **Table S3 List of ORFs identified in the genome of phage SL3*** | |
| --- | --- | --- |
|  |  |  |
| 1 | 276..1817 | PHAGE_PaeP_C2_10_Ab22_NC_026599: hypoth. protein; PP_00001; phage(gi764161091) |
| 2 | 1814..2503 | PHAGE_PaeP_C2_10_Ab22_NC_026599: hypoth. protein; PP_00002; phage(gi764161090) |
| 3 | 2500..2934 | PHAGE_PaeP_C2_10_Ab22_NC_026599: hypoth. protein; PP_00003; phage(gi764161089) |
| 4 | 2915..3853 | PHAGE_PaeP_C2_10_Ab22_NC_026599: hypoth. protein; PP_00004; phage(gi764161088) |
| 5 | 4238..5755 | PHAGE_PaeP_C2_10_Ab22_NC_026599: hypoth. protein; PP_00005; phage(gi764161086) |
| 6 | 5906..9073 | PHAGE_PaeP_C2_10_Ab22_NC_026599: hypoth. protein; PP_00006; phage(gi764161085) |
| 7 | 9085..9972 | PHAGE_PaeP_C2_10_Ab22_NC_026599: hypoth. protein; PP_00007; phage(gi764161084) |
| 8 | 9986..10342 | PHAGE_PaeP_C2_10_Ab22_NC_026599: hypoth. protein; PP_00008; phage(gi764161083) |
| 9 | c(10386..10613) | PHAGE_PaeP_C2_10_Ab22_NC_026599: hypoth. protein; PP_00009; phage(gi764161082) |
| 10 | c(10758..10964) | PHAGE_PaeP_C2_10_Ab22_NC_026599: hypoth. protein; PP_00010; phage(gi764161081) |
| 11 | c(10951..11160) | PHAGE_PaeP_C2_10_Ab22_NC_026599: hypoth. protein; PP_00011; phage(gi764161080) |
| 12 | c(11164..11382) | PHAGE_PaeP_C2_10_Ab22_NC_026599: hypoth. protein; PP_00012; phage(gi764161079) |
| 13 | c(11379..12140) | PHAGE_PaeP_C2_10_Ab22_NC_026599: hypoth. protein; PP_00013; phage(gi764161078) |
| 14 | c(12133..12372) | PHAGE_PaeP_C2_10_Ab22_NC_026599: hypoth. protein; PP_00014; phage(gi764161077) |
| 15 | c(12335..13318) | PHAGE_PaeP_C2_10_Ab22_NC_026599: hypoth. protein; PP_00015; phage(gi764161076) |
| 16 | c(13293..14177) | PHAGE_PaeP_C2_10_Ab22_NC_026599: put. 5'-3' exonuclease; PP_00016; phage(gi764161075) |
| 17 | c(14177..14461) | PHAGE_PaeP_C2_10_Ab22_NC_026599: hypoth. protein; PP_00017; phage(gi764161073) |
| 18 | c(14433..14828) | PHAGE_PaeP_C2_10_Ab22_NC_026599: hypoth. protein; PP_00018; phage(gi764161072) |
| 19 | c(14914..15474) | PHAGE_PaeP_C2_10_Ab22_NC_026599: hypoth. protein; PP_00019; phage(gi764161071) |
| 20 | c(15544..17181) | PHAGE_PaeP_C2_10_Ab22_NC_026599: put. DNA polymerase; PP_00020; phage(gi764161070) |
| 21 | c(17182..17379) | PHAGE_PaeP_C2_10_Ab22_NC_026599: hypoth. protein; PP_00021; phage(gi764161069) |
| 22 | c(17384..17893) | PHAGE_PaeP_C2_10_Ab22_NC_026599: hypoth. protein; PP_00022; phage(gi764161068) |
| 23 | c(17970..18248) | PHAGE_PaeP_C2_10_Ab22_NC_026599: put. holin; PP_00023; phage(gi764161067) |
| 24 | c(18245..18664) | PHAGE_LUZ24_NC_010325: hypoth. protein; PP_00024; phage(gi167600403) |
| 25 | c(18655..18816) | PHAGE_PaeP_C2_10_Ab22_NC_026599: hypoth. protein; PP_00025; phage(gi764161066) |
| 26 | c(18806..18955) | PHAGE_PaeP_C2_10_Ab22_NC_026599: hypoth. protein; PP_00026; phage(gi764161065) |
| 27 | c(18978..19211) | PHAGE_PaeP_C2_10_Ab22_NC_026599: hypoth. protein; PP_00027; phage(gi764161064) |
| 28 | c(19247..19543) | PHAGE_PaeP_C2_10_Ab22_NC_026599: hypoth. protein; PP_00028; phage(gi764161063) |
| 29 | c(19525..19896) | PHAGE_PaeP_C2_10_Ab22_NC_026599: put. DNA polymerase; PP_00030; phage(gi764161062) |
| 30 | 19873..20001 | hypoth.; PP_00029 |
| 31 | c(20018..21649) | PHAGE_PaeP_C2_10_Ab22_NC_026599: put. DNA primase/helicase; PP_00031; phage(gi764161061) |
| 32 | c(21728..22105) | PHAGE_PaeP_C2_10_Ab22_NC_026599: hypoth. protein; PP_00032; phage(gi764161060) |
| 33 | c(22105..22503) | PHAGE_PaeP_C2_10_Ab22_NC_026599: hypoth. protein; PP_00033; phage(gi764161059) |
| 34 | c(22503..23387) | PHAGE_PaeP_C2_10_Ab22_NC_026599: hypoth. protein; PP_00034; phage(gi764161058) |
| 35 | c(23534..23755) | PHAGE_PaeP_C2_10_Ab22_NC_026599: hypoth. protein; PP_00035; phage(gi764161057) |
| 36 | c(23765..25297) | PaeP_C2_10_Ab22_NC_026599: L-glutamine-D-fructose-6-phosph. amidotransferase; PP36(gi764161056) |
| 37 | c(25309..26484) | PHAGE_PaeP_C2_10_Ab22_NC_026599: hypoth. protein; PP_00037; phage(gi764161055) |
| 38 | c(26460..27029) | PHAGE_PaeP_C2_10_Ab22_NC_026599: hypoth. protein; PP_00038; phage(gi764161054) |
| 39 | c(27022..27822) | PHAGE_PaeP_C2_10_Ab22_NC_026599: hypoth. protein; PP_00039; phage(gi764161053) |
| 40 | c(27819..28778) | PHAGE_PaeP_C2_10_Ab22_NC_026599: hypoth. protein; PP_00040; phage(gi764161052) |
| 41 | c(28797..29759) | PHAGE_PaeP_C2_10_Ab22_NC_026599: hypoth. protein; PP_00041; phage(gi764161051) |
| 42 | c(29830..29958) | PHAGE_PaeP_C2_10_Ab22_NC_026599: hypoth. protein; PP_00042; phage(gi764161050) |
| 43 | c(29970..30362) | PHAGE_PaeP_C2_10_Ab22_NC_026599: hypoth. protein; PP_00043; phage(gi764161045) |
| 44 | c(30604..30771) | PHAGE_DL54_NC_028919: hypoth. protein; PP_00044; phage(gi971755623) |
| 45 | c(30771..31052) | PHAGE_PhiCHU_NC_028933: hypoth. protein; PP_00045; phage(gi971757276) |
| 46 | c(31442..31597) | PHAGE_PaeP_C2_10_Ab22_NC_026599: hypoth. protein; PP_00046; phage(gi764161040) |
| 47 | 31621..31818 | hypoth.; PP_00047 |
| 48 | c(31993..32175) | PHAGE_PaeP_C2_10_Ab22_NC_026599: hypoth. protein; PP_00048; phage(gi764161038) |
| 49 | c(32355..32495) | PHAGE_PaeP_C2_10_Ab22_NC_026599: hypoth. protein; PP_00049; phage(gi764161037) |
| 50 | c(32779..32922) | PHAGE_PaeP_C2_10_Ab22_NC_026599: hypoth. protein; PP_00050; phage(gi764161035) |
| 51 | c(32922..33476) | PHAGE_PaeP_C2_10_Ab22_NC_026599: hypoth. protein; PP_00051; phage(gi764161034) |
| 52 | c(33494..33664) | PHAGE_PaeP_C2_10_Ab22_NC_026599: hypoth. protein; PP_00052; phage(gi764161033) |
| 53 | c(34682..34804) | hypoth.; PP_00053 |
| 54 | 35378..35453 | tRNA |
| 55 | 35550..35626 | tRNA |
| 56 | 35649..36107 | PHAGE_PaeP_C2_10_Ab22_NC_026599: hypoth. protein; PP_00054; phage(gi764161103) |
| 57 | 36139..36537 | PHAGE_PaeP_C2_10_Ab22_NC_026599: put. baseplate hub protein; PP_00055; phage(gi764161102) |
| 58 | 36537..37985 | PHAGE_PaeP_C2_10_Ab22_NC_026599: put. terminase, large subunit; PP_00056; phage(gi764161101) |
| 59 | 37985..40105 | PHAGE_PaeP_C2_10_Ab22_NC_026599: put. portal protein; PP_00057; phage(gi764161100) |
| 60 | 40108..40350 | PHAGE_PaeP_C2_10_Ab22_NC_026599: hypoth. protein; PP_00058; phage(gi764161099) |
| 61 | 40350..41342 | PHAGE_PaeP_C2_10_Ab22_NC_026599: put. capsid and scaffold protein; PP_00059; phage(gi764161098) |
| 62 | 41361..42314 | PHAGE_PaeP_C2_10_Ab22_NC_026599: put. capsid and scaffold protein; PP_00060; phage(gi764161097) |
| 63 | 42363..42683 | PHAGE_PaeP_C2_10_Ab22_NC_026599: hypoth. protein; PP_00061; phage(gi764161096) |
| 64 | 42687..43313 | PHAGE_PaeP_C2_10_Ab22_NC_026599: hypoth. protein; PP_00062; phage(gi764161095) |
| 65 | 43324..43515 | PHAGE_PaeP_C2_10_Ab22_NC_026599: hypoth. protein; PP_00063; phage(gi764161094) |
|  | **c = complement*** |  |
|  |  |  |
|  | **Summary** | 52 hypothetical proteins |
|  |  | 2 tRNAs |
|  |  | 5 genes for host-independant DNA replication machinery |
|  |  | 4 structural proteins |
|  |  | 1 holin |
|  |  | 1 L-glutamine-D-fructose-6-phosph. amidotransferase |
|  |  | In total 65 ORFS |
