# Supplementary material for: Differential Effect of Newly Isolated Phages Belonging to PB1-Like, phiKZ-Like and LUZ24-Like Viruses against Multi-Drug Resistant Pseudomonas aeruginosa under Varying Growth Conditions
Source: Viruses. 2017 Oct 27;9(11):315. doi: 10.3390/v9110315 (PMC5707522; doi:10.3390/v9110315)
Supplement: Supplementary file 1 [file viruses-09-00315-s001.zip › Table S4.docx]

**Table S4 Studies investigating the antibacterial activity of phages against P. aeruginosa^1,2^**

**^1^** Studies testing host range only and studies without genome-based classification of phages are not included; the list may not be complete; **^2^** blue circles: *in vitro* assays, red circles: *in vivo*/*ex vivo* studies

References

[1. Saussereau, E.; Vachier, I.; Chiron, R.; Godbert, B.; Sermet, I.; Dufour, N.; Pirnay, J.-P.; De Vos, D.; Carrié, F.; Molinari, N.; Debarbieux, L. Effectiveness of bacteriophages in the sputum of cystic fibrosis patients. *Clin. Microbiol. Infect.* **2014**, *20*, O983–O990, doi:10.1111/1469-0691.12712.

2. Henry, M.; Lavigne, R.; Debarbieux, L. Predicting In Vivo Efficacy of Therapeutic Bacteriophages Used To Treat Pulmonary Infections. *Antimicrob. Agents Chemother.* **2013**, *57*, 5961–5968, doi:10.1128/AAC.01596-13.

3. Alves, D. R.; Perez-Esteban, P.; Kot, W.; Bean, J. E.; Arnot, T.; Hansen, L. H.; Enright, M. C.; Jenkins, A. T. A. A novel bacteriophage cocktail reduces and disperses *P* *seudomonas aeruginosa* biofilms under static and flow conditions: Bacteriophages to treat *P. aeruginosa* biofilms. *Microb. Biotechnol.* **2016**, *9*, 61–74, doi:10.1111/1751-7915.12316.

4. Betts, A.; Kaltz, O.; Hochberg, M. E. Contrasted coevolutionary dynamics between a bacterial pathogen and its bacteriophages. *Proc. Natl. Acad. Sci.* **2014**, *111*, 11109–11114, doi:10.1073/pnas.1406763111.

5. Hall, A. R.; De Vos, D.; Friman, V.-P.; Pirnay, J.-P.; Buckling, A. Effects of Sequential and Simultaneous Applications of Bacteriophages on Populations of Pseudomonas aeruginosa In Vitro and in Wax Moth Larvae. *Appl. Environ. Microbiol.* **2012**, *78*, 5646–5652, doi:10.1128/AEM.00757-12.

6. Friman, V.-P.; Soanes-Brown, D.; Sierocinski, P.; Molin, S.; Johansen, H. K.; Merabishvili, M.; Pirnay, J.-P.; De Vos, D.; Buckling, A. Pre-adapting parasitic phages to a pathogen leads to increased pathogen clearance and lowered resistance evolution with *Pseudomonas aeruginosa* cystic fibrosis bacterial isolates. *J. Evol. Biol.* **2016**, *29*, 188–198, doi:10.1111/jeb.12774.

7. Betts, A.; Gifford, D. R.; MacLean, R. C.; King, K. C. Parasite diversity drives rapid host dynamics and evolution of resistance in a bacteria-phage system: GROWTH AND RESISTANCE WITH PARASITE DIVERSITY. *Evolution* **2016**, *70*, 969–978, doi:10.1111/evo.12909.

8. Mapes, A. C.; Trautner, B. W.; Liao, K. S.; Ramig, R. F. Development of expanded host range phage active on biofilms of multi-drug resistant *Pseudomonas aeruginosa*. *Bacteriophage* **2016**, *6*, e1096995, doi:10.1080/21597081.2015.1096995.

9. Kwiatek, M.; Parasion, S.; Rutyna, P.; Mizak, L.; Gryko, R.; Niemcewicz, M.; Olender, A.; Łobocka, M. Isolation of bacteriophages and their application to control Pseudomonas aeruginosa in planktonic and biofilm models. *Res. Microbiol.* **2017**, *168*, 194–207, doi:10.1016/j.resmic.2016.10.009.

10. Furusawa, T.; Iwano, H.; Hiyashimizu, Y.; Matsubara, K.; Higuchi, H.; Nagahata, H.; Niwa, H.; Katayama, Y.; Kinoshita, Y.; Hagiwara, K.; Iwasaki, T.; Tanji, Y.; Yokota, H.; Tamura, Y. Phage Therapy Is Effective in a Mouse Model of Bacterial Equine Keratitis. *Appl. Environ. Microbiol.* **2016**, *82*, 5332–5339, doi:10.1128/AEM.01166-16.

11. Mendes, J. J.; Leandro, C.; Mottola, C.; Barbosa, R.; Silva, F. A.; Oliveira, M.; Vilela, C. L.; Melo-Cristino, J.; Gorski, A.; Pimentel, M.; Sao-Jose, C.; Cavaco-Silva, P.; Garcia, M. In vitro design of a novel lytic bacteriophage cocktail with therapeutic potential against organisms causing diabetic foot infections. *J. Med. Microbiol.* **2014**, *63*, 1055–1065, doi:10.1099/jmm.0.071753-0.

12. Alemayehu, D.; Casey, P. G.; McAuliffe, O.; Guinane, C. M.; Martin, J. G.; Shanahan, F.; Coffey, A.; Ross, R. P.; Hill, C. Bacteriophages MR299-2 and NH-4 Can Eliminate Pseudomonas aeruginosa in the Murine Lung and on Cystic Fibrosis Lung Airway Cells. *mBio* **2012**, *3*, e00029–12–e00029–12, doi:10.1128/mBio.00029-12.

13. Darch, S. E.; Kragh, K. N.; Abbott, E. A.; Bjarnsholt, T.; Bull, J. J.; Whiteley, M. Phage Inhibit Pathogen Dissemination by Targeting Bacterial Migrants in a Chronic Infection Model. *mBio* **2017**, *8*, e00240–17, doi:10.1128/mBio.00240-17.

14. Olszak, T.; Zarnowiec, P.; Kaca, W.; Danis-Wlodarczyk, K.; Augustyniak, D.; Drevinek, P.; de Soyza, A.; McClean, S.; Drulis-Kawa, Z. In vitro and in vivo antibacterial activity of environmental bacteriophages against Pseudomonas aeruginosa strains from cystic fibrosis patients. *Appl. Microbiol. Biotechnol.* **2015**, *99*, 6021–6033, doi:10.1007/s00253-015-6492-6.

15. Chaudhry, W. N.; Concepción-Acevedo, J.; Park, T.; Andleeb, S.; Bull, J. J.; Levin, B. R. Synergy and Order Effects of Antibiotics and Phages in Killing Pseudomonas aeruginosa Biofilms. *PLOS ONE* **2017**, *12*, e0168615, doi:10.1371/journal.pone.0168615.

16. Uchiyama, J.; Suzuki, M.; Nishifuji, K.; Kato, S.; Miyata, R.; Nasukawa, T.; Yamaguchi, K.; Takemura-Uchiyama, I.; Ujihara, T.; Shimakura, H.; Murakami, H.; Okamoto, N.; Sakaguchi, Y.; Shibayama, K.; Sakaguchi, M.; Matsuzaki, S. Analyses of Short-Term Antagonistic Evolution of Pseudomonas aeruginosa Strain PAO1 and Phage KPP22 (Myoviridae Family, PB1-Like Virus Genus). *Appl. Environ. Microbiol.* **2016**, *82*, 4482–4491, doi:10.1128/AEM.00090-16.

17. Morello, E.; Saussereau, E.; Maura, D.; Huerre, M.; Touqui, L.; Debarbieux, L. Pulmonary Bacteriophage Therapy on Pseudomonas aeruginosa Cystic Fibrosis Strains: First Steps Towards Treatment and Prevention. *PLoS ONE* **2011**, *6*, e16963, doi:10.1371/journal.pone.0016963.

18. Danis-Wlodarczyk, K.; Olszak, T.; Arabski, M.; Wasik, S.; Majkowska-Skrobek, G.; Augustyniak, D.; Gula, G.; Briers, Y.; Jang, H. B.; Vandenheuvel, D.; Duda, K. A.; Lavigne, R.; Drulis-Kawa, Z. Characterization of the Newly Isolated Lytic Bacteriophages KTN6 and KT28 and Their Efficacy against Pseudomonas aeruginosa Biofilm. *PLOS ONE* **2015**, *10*, e0127603, doi:10.1371/journal.pone.0127603.

19. Torres-Barceló, C.; Franzon, B.; Vasse, M.; Hochberg, M. E. Long-term effects of single and combined introductions of antibiotics and bacteriophages on populations of *Pseudomonas aeruginosa*. *Evol. Appl.* **2016**, *9*, 583–595, doi:10.1111/eva.12364.

20. Torres-Barceló, C.; Arias-Sánchez, F. I.; Vasse, M.; Ramsayer, J.; Kaltz, O.; Hochberg, M. E. A Window of Opportunity to Control the Bacterial Pathogen Pseudomonas aeruginosa Combining Antibiotics and Phages. *PLoS ONE* **2014**, *9*, e106628, doi:10.1371/journal.pone.0106628.

21. Vieira, A.; Silva, Y. J.; Cunha, â.; Gomes, N. C. M.; Ackermann, H.-W.; Almeida, A. Phage therapy to control multidrug-resistant Pseudomonas aeruginosa skin infections: in vitro and ex vivo experiments. *Eur. J. Clin. Microbiol. Infect. Dis.* **2012**, *31*, 3241–3249, doi:10.1007/s10096-012-1691-x.

22. Watanabe, R.; Matsumoto, T.; Sano, G.; Ishii, Y.; Tateda, K.; Sumiyama, Y.; Uchiyama, J.; Sakurai, S.; Matsuzaki, S.; Imai, S.; Yamaguchi, K. Efficacy of Bacteriophage Therapy against Gut-Derived Sepsis Caused by Pseudomonas aeruginosa in Mice. *Antimicrob. Agents Chemother.* **2007**, *51*, 446–452, doi:10.1128/AAC.00635-06.

23. Debarbieux, L.; Leduc, D.; Maura, D.; Morello, E.; Criscuolo, A.; Grossi, O.; Balloy, V.; Touqui, L. Bacteriophages Can Treat and Prevent *Pseudomonas aeruginosa* Lung Infections. *J. Infect. Dis.* **2010**, *201*, 1096–1104, doi:10.1086/651135.

24. Danis-Wlodarczyk, K.; Vandenheuvel, D.; Jang, H. B.; Briers, Y.; Olszak, T.; Arabski, M.; Wasik, S.; Drabik, M.; Higgins, G.; Tyrrell, J.; Harvey, B. J.; Noben, J.-P.; Lavigne, R.; Drulis-Kawa, Z. A proposed integrated approach for the preclinical evaluation of phage therapy in Pseudomonas infections. *Sci. Rep.* **2016**, *6*, doi:10.1038/srep28115.

25. Chan, B. K.; Sistrom, M.; Wertz, J. E.; Kortright, K. E.; Narayan, D.; Turner, P. E. Phage selection restores antibiotic sensitivity in MDR Pseudomonas aeruginosa. *Sci. Rep.* **2016**, *6*, doi:10.1038/srep26717.

26. Fukuda, K.; Ishida, W.; Uchiyama, J.; Rashel, M.; Kato, S.; Morita, T.; Muraoka, A.; Sumi, T.; Matsuzaki, S.; Daibata, M.; Fukushima, A. Pseudomonas aeruginosa Keratitis in Mice: Effects of Topical Bacteriophage KPP12 Administration. *PLoS ONE* **2012**, *7*, e47742, doi:10.1371/journal.pone.0047742.

27. Kim, S.; Rahman, M.; Seol, S. Y.; Yoon, S. S.; Kim, J. Pseudomonas aeruginosa Bacteriophage PA1O Requires Type IV Pili for Infection and Shows Broad Bactericidal and Biofilm Removal Activities. *Appl. Environ. Microbiol.* **2012**, *78*, 6380–6385, doi:10.1128/AEM.00648-12.

28. Kay, M. K.; Erwin, T. C.; McLean, R. J. C.; Aron, G. M. Bacteriophage Ecology in Escherichia coli and Pseudomonas aeruginosa Mixed-Biofilm Communities. *Appl. Environ. Microbiol.* **2011**, *77*, 821–829, doi:10.1128/AEM.01797-10.

29. Garbe, J.; Wesche, A.; Bunk, B.; Kazmierczak, M.; Selezska, K.; Rohde, C.; Sikorski, J.; Rohde, M.; Jahn, D.; Schobert, M. Characterization of JG024, a pseudomonas aeruginosa PB1-like broad host range phage under simulated infection conditions. *BMC Microbiol.* **2010**, *10*, 301, doi:10.1186/1471-2180-10-301.

30. Tiwari, B. R.; Kim, S.; Rahman, M.; Kim, J. Antibacterial efficacy of lytic Pseudomonas bacteriophage in normal and neutropenic mice models. *J. Microbiol.* **2011**, *49*, 994–999, doi:10.1007/s12275-011-1512-4.

31. Yang, M.; Du, C.; Gong, P.; Xia, F.; Sun, C.; Feng, X.; Lei, L.; Song, J.; Zhang, L.; Wang, B.; Xiao, F.; Yan, X.; Cui, Z.; Li, X.; Gu, J.; Han, W. Therapeutic effect of the YH6 phage in a murine hemorrhagic pneumonia model. *Res. Microbiol.* **2015**, *166*, 633–643, doi:10.1016/j.resmic.2015.07.008.

32. Cao, Z.; Zhang, J.; Niu, Y. D.; Cui, N.; Ma, Y.; Cao, F.; Jin, L.; Li, Z.; Xu, Y. Isolation and Characterization of a “phiKMV-Like” Bacteriophage and Its Therapeutic Effect on Mink Hemorrhagic Pneumonia. *PLOS ONE* **2015**, *10*, e0116571, doi:10.1371/journal.pone.0116571.

33. Coulter, L.; McLean, R.; Rohde, R.; Aron, G. Effect of Bacteriophage Infection in Combination with Tobramycin on the Emergence of Resistance in Escherichia coli and Pseudomonas aeruginosa Biofilms. *Viruses* **2014**, *6*, 3778–3786, doi:10.3390/v6103778.

34. Lim, W. S.; Phang, K. K. S.; Tan, A. H.-M.; Li, S. F.-Y.; Ow, D. S.-W. Small Colony Variants and Single Nucleotide Variations in Pf1 Region of PB1 Phage-Resistant Pseudomonas aeruginosa. *Front. Microbiol.* **2016**, *7*, doi:10.3389/fmicb.2016.00282.
