# Supplementary material for: Differential Effect of Newly Isolated Phages Belonging to PB1-Like, phiKZ-Like and LUZ24-Like Viruses against Multi-Drug Resistant Pseudomonas aeruginosa under Varying Growth Conditions
Source: Viruses. 2017 Oct 27;9(11):315. doi: 10.3390/v9110315 (PMC5707522; doi:10.3390/v9110315)
Supplement: Supplementary file 1 [file viruses-09-00315-s001.zip › Supplementary Informatioon S1.docx]

Article

Differential Effect of Newly Isolated Phages Belonging to PB1-Like, phiKZ-Like and LUZ24-Like Viruses against Multi-Drug Resistant Pseudomonas aeruginosa under Varying Growth Conditions

Simone Latz, Alex Krüttgen, Helga Häfner, Eva Miriam Buhl, Klaus Ritter, Hans-Peter Horz

Supplementary information

Phage SL1 (65,849 bp) shows a genome wide nucleotide sequence similarity of 94 to 96% to members of the PB1-like viruses (Table 1 and Fig. S2). The GC content is 55.56% and no tRNA coding genes are present in the genome. In total, 91 open reading frames (ORFs) were identified of which 64 (70.3%) were designated as hypothetical proteins (Supplemental Table S1). 20 ORFs (22%) were identified as structural proteins (13 with no further specifications, the others encoding for two putative minor head proteins, one capsid and scaffold protein, one putative lytic tail protein, two putative baseplate proteins, and one tail fibers protein).

Further genome contents include genes relevant for a host-independent DNA replication machinery, such as a helicase, a DNA polymerase III, a thymidylate synthase, an exonuclease, a primase, and a terminase. In accordance with PB1-like viruses the genome does not contain genes encoding for an RNA polymerase suggesting that phage SL1 uses the host transcriptional machinery.

Phage SL2 (a giant phage with 279,696 bp) shows a genome wide nucleotide sequence similarity of 69 - 99% to members of the phiKZ-like viruses, with phages phiKZ and KTN4 being the closest relatives (Table 1, Fig. S3). The GC content is 36.9% and five tRNAs, Leu (UUA), Pro (UUG), 2 x Asn (GUU) and Thr (UGU) are present in the genome. In total 355 ORFs were identified of which 344 ORFs (97%) were assigned as hypothetical proteins using PHAST, while four structural proteins and one homing endonuclease of the GIY-YIG family were identified (Supplemental Table S2). More detailed genome information was obtained by comparing the deduced protein sequences of the 355 ORFs with the genome of KTN4 from which a structural proteome analysis (ESI-MS/MS) had previously led to the identification of 111 virion-associated gene products [Danis-Wlodarczyk, et al. 2016]. Phage SL2 shares a sequence identity of 98-100% at protein level with those annotated gene products of KTN4 except for the Thymidylate synthase gene (77% sequence identity at amino acid level).

Phage SL4 (44,194 bp) shows a genome wide nucleotide sequence similarity of 91 - 95% to the five most closely related members of the LUZ24-like viruses (Table 1, Fig. S4). The GC content is 52.3% and two tRNA coding genes were identified, Asn (GUU), Tyr (GUA). In total 65 ORFs were identified of which 52 (80%) were designated as hypothetical proteins (Supplemental Table S3). Structural proteins included one baseplate hub protein, one portal protein, and two capsid and scaffold proteins. Further genes of known functions included those relevant for DNA metabolism, such as a primase/helicase, two DNA polymerases, a 5'-3' exonuclease, and a terminase. In addition, one gene encoding for a member of the holin gene family and one gene encoding for the L-glutamine-D-fructose-6-phosphate amidotransferase were identified.

Reference

Danis-Wlodarczyk, et al. A proposed integrated approach for the preclinical evaluation of phage therapy in Pseudomonas infections. Sci. Rep. 2016, 6, doi:10.1038/srep28115
